# CXCL1 mediates obesity-associated adipose stromal cell trafficking and function in the tumour microenvironment

**DOI:** 10.1038/ncomms11674

**Published:** 2016-05-31

**Authors:** Tao Zhang, Chieh Tseng, Yan Zhang, Olga Sirin, Paul G. Corn, Elsa M. Li-Ning-Tapia, Patricia Troncoso, John Davis, Curtis Pettaway, John Ward, Marsha L. Frazier, Christopher Logothetis, Mikhail G. Kolonin

**Affiliations:** 1 The Brown Foundation Institute of Molecular Medicine, University of Texas Health Science Center at Houston, Houston, Texas 77030, USA; 2 Department of Genitourinary Medical Oncology, The University of Texas MD Anderson Cancer Center, Houston, Texas 77030, USA; 3 Department of Pathology, The University of Texas MD Anderson Cancer Center, Houston, Texas 77030, USA; 4 Department of Epidemiology, The University of Texas MD Anderson Cancer Center, Houston, Texas 77030, USA

## Abstract

White adipose tissue (WAT) overgrowth in obesity is linked with increased aggressiveness of certain cancers. Adipose stromal cells (ASCs) can become mobilized from WAT, recruited by tumours and promote cancer progression. Mechanisms underlying ASC trafficking are unclear. Here we demonstrate that chemokines CXCL1 and CXCL8 chemoattract ASC by signalling through their receptors, CXCR1 and CXCR2, in cell culture models. We further show that obese patients with prostate cancer have increased epithelial CXCL1 expression. Concomitantly, we observe that cells with ASC phenotype are mobilized and infiltrate tumours in obese patients. Using mouse models, we show that the CXCL1 chemokine gradient is required for the obesity-dependent tumour ASC recruitment, vascularization and tumour growth promotion. We demonstrate that αSMA expression in ASCs is induced by chemokine signalling and mediates the stimulatory effects of ASCs on endothelial cells. Our data suggest that ASC recruitment to tumours, driven by CXCL1 and CXCL8, promotes prostate cancer progression.

## Abstract (web summary)

Adipose stromal cells (ASC) have been shown to migrate to tumours and promote tumour growth. Using animal models and human tissue samples, the authors show here that ASC recruitment to prostate cancers is mediated by the chemokine CXCL1, which is secreted from tumour cells, and acts on CXCR1 on ASCs.

## Graphical abstract

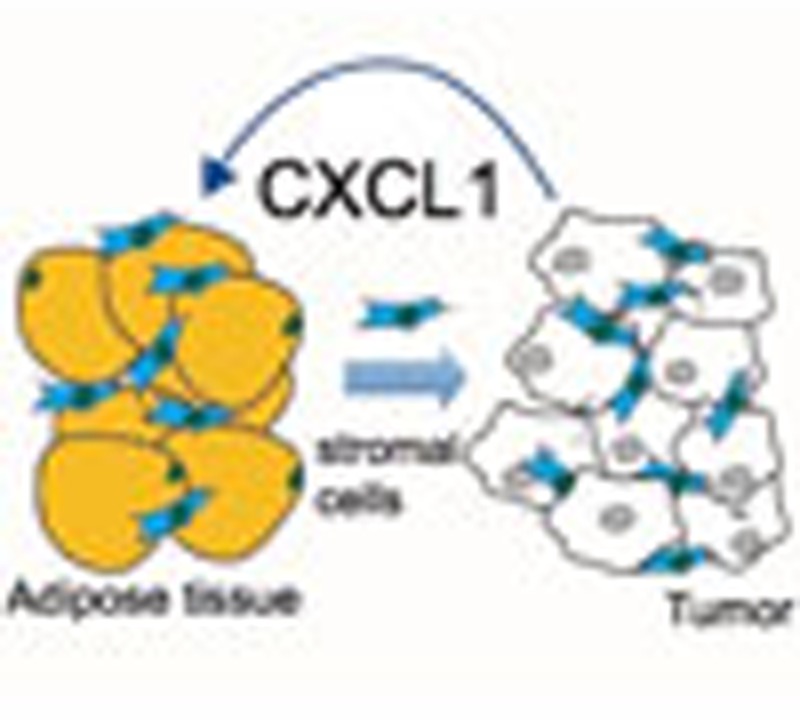

The tumour microenvironment is one of the determinants of cancer progression^1^. Tumour stroma, dynamically changing during cancer progression, is composed of a number of cell populations, aetiology of which is incompletely understood^2^,^3^. While the pool of tumour leukocytes, such as myeloid-derived suppressor cells (MDSCs), is maintained by haematopoietic progenitors^4^,^5^,^6^,^7^, the cancer-associated fibroblasts (CAFs) are of mesenchymal origin^8^,^9^,^10^. Some of the mesenchymal cancer stroma may be derived from prostate-resident cells^11^; however, recruitment of mesenchymal stromal cells (MSCs) from other tissues is also documented^6^,^12^,^13^. Mesenchymal stroma influences distinct stages of cancer progression and resistance to therapy through the complex mechanisms^14^,^15^. MSCs secrete tumour growth factor-beta, a cytokine implicated in the epithelial–mesenchymal transition, and a plethora of other angiogenic, immunosuppressive, anti-apoptotic and mitogenic factors^12^,^16^. MSCs promote tumour vascularization and are responsible for deposition of extracellular matrix and tumour desmoplasia^17^. They can also mute anti-tumour immune response through their effect on T cells and tumour-associated macrophages, which are also key players in cancer progression^7^,^18^.

While monocytes and lymphocytes found in tumour stroma originate from the bone marrow, accumulating data demonstrate that mesenchymal CAFs are also recruited from extramedullary organs^19^,^20^. Indeed, relatively low numbers of MSCs are found in the bone marrow, while some other organs have been revealed as key MSC reservoirs. One of the organs harbouring MSCs capable of stimulating tumours is white adipose tissue (WAT), which is overgrown in obese individuals^14^,^21^. A number of epidemiological studies have provided evidence that the progression of prostate cancer is associated with obesity^22^,^23^,^24^. Increased body mass index (BMI), waist-to-hip ratio (an indicator of abdominal adiposity), as well as overgrowth of periprostatic (PP) WAT are associated with more aggressive tumours and adverse outcome, including mortality^25^,^26^. The biological connection between cancer and obesity is complex and incompletely understood^21^. As the prevalence of obesity is rising, insights into the mechanisms underlying its link with cancer aggressiveness are urgently needed to develop new strategies for reducing prostate cancer morbidity and mortality.

Studies in mouse models have shown that WAT overgrowth is sufficient to enhance cancer progression irrespective of diet^27^. Trophic factors released by cells of WAT may account for that effect. Monocytes/macrophages and other WAT-infiltrating leukocytes, as well as adipocytes and their mesenchymal progenitors termed adipose stromal cells (ASCs), secrete hormones, cytokines and growth factors collectively termed adipokines^28^. Proliferation of ASCs, the WAT-resident MSCs, accompanies WAT expansion^27^. In a series of studies, we have shown that in obesity increased numbers of ASCs migrate from WAT and contribute to tumour microenvironment^27^,^29^,^30^. Mobilization of ASCs into the peripheral blood has been reported in human obesity and is further elevated in cancer patients^31^, which suggests systemic circulation as a route of ASC trafficking to tumours. In animal models, transplanted ASCs migrate to tumours, engraft and promote tumour growth^27^,^29^,^30^. Our findings, confirmed by the data from other laboratories^20^,^32^,^33^, suggest that ASCs facilitate tumour vascularization, which enables increased survival and proliferation of neighbouring malignant cells and, hence, cancer progression^34^. The capacity of ASCs to promote metastatic dissemination has also been reported^32^,^33^.

Hypoxia and inflammation signals have been proposed to guide MSC trafficking to tumours; however, specific signalling events remain unknown^14^. Migration of cells in the body is directed by chemokine gradients^35^,^36^. Our previous studies showed that human endometrial cancer cells secrete chemokines (C–X–C motif) ligand 1 (CXCL1), also known as KC and GROα, as well as a related chemokine CXCL8 (also known as interleukin-8)^30^. These two chemokines serve as ligands of chemokine receptors CXCR1 and CXCR2, which we reported to be expressed in human ASCs^30^,^37^. CXCL1 overexpression in mouse epithelium induces prostate hyperplasia and reactive stroma, recruitment of which accompanies the progression of human disease^38^. A recent report demonstrated that CXCL1 expression is increased in high-grade prostate cancer^39^, warranting studies on the function of its signalling in disease progression.

Here we demonstrate that both PP and subcutaneous (SC) ASCs migrate towards CXCL1 and CXCL8 by signalling via their receptors CXCR1 and CXCR2. We show that in prostate cancer CXCL1 expression is obesity dependent, while CXCL8 expression is obesity independent in malignant tumour cells. Concordantly, obese patients tend to have cells with ASC properties in the systemic circulation. We show that the chemokine receptor CXCR1 is expressed by ASCs *in vivo*, and that in obese patients, tumour stroma is infiltrated by CXCR1-positive cells. Using murine cancer models, we demonstrate that the CXCL1 signalling through its receptors is rate-limiting for the obesity-associated ASC recruitment to tumours and their stimulatory effects on tumour vascularization and growth. Our data indicate that CXCR1 signalling induces α-smooth muscle actin (αSMA) expression in ASCs and their stimulatory effects on endothelial cells, thus suggesting the molecular mechanism through which ASCs are recruited by tumours and promote their vascularization and growth.

## Results

### Stromal cell mobilization in obese prostate cancer patients

**Figure 1:**
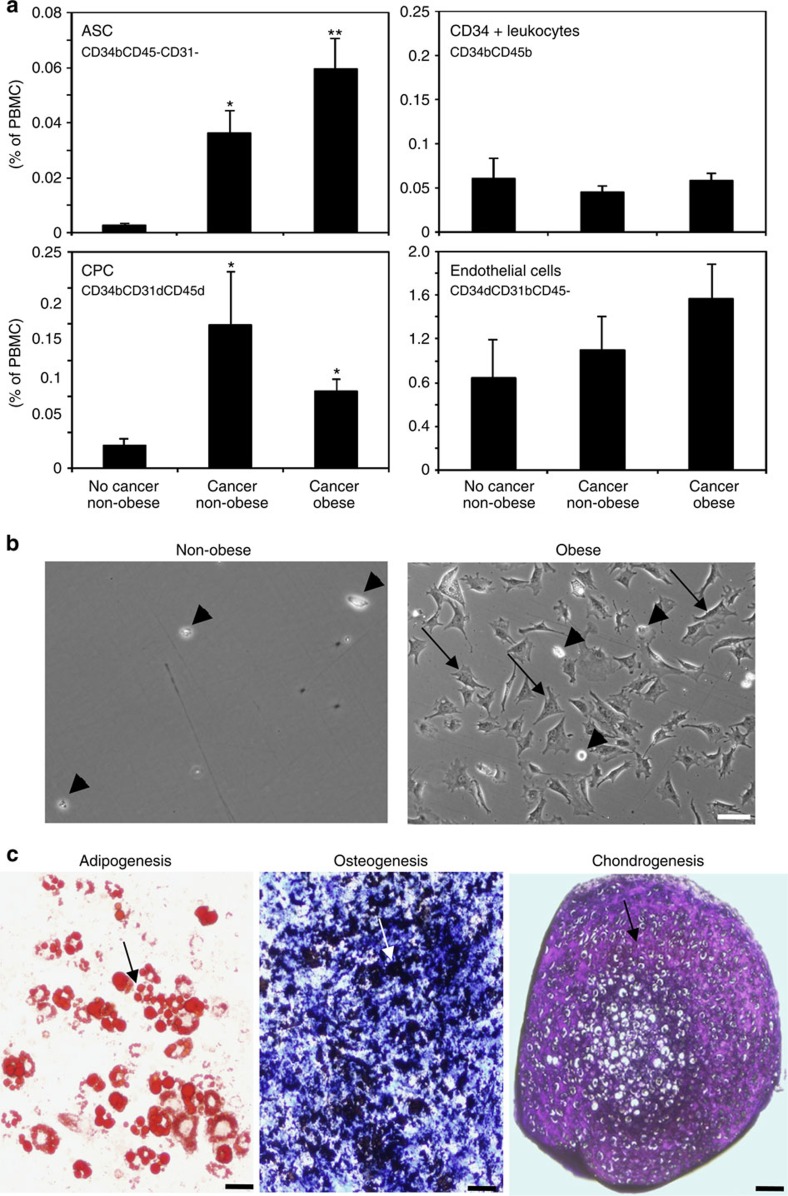
Increased ASC circulation in obese prostate cancer patients. (**a**) Flow cytometric enumeration of CD34brightCD45−CD31− ASCs, CD34brightCD45bright leukocytes, CD34brightCD31dimCD45dim CPCs (EPC/HPC) and CD34dimCD31brightCD45− endothelial cells in PBMCs of 12 healthy non-obese donors versus 24 non-obese and 21 obese prostate cancer patients. Graphs show mean±s.e.m.; *P<0.05 versus cancer-free non-obese donor; **P<0.05 versus non-obese cancer patient (Mann–Whitney *U*-test). (**b**) Representative bright-field micrographs of adherent cells recovered from equal numbers of PBMCs from representative non-obese and obese prostate cancer patients. Arrowheads: cells with monocyte morphology, arrows: cells with ASC morphology. (**c**) Three-lineage differentiation of obese patient PBMC-derived stromal cells expanded in culture (**b**). For >70% of culture area analysed, adipogenesis is evident from Oil red O staining of lipid droplets (red); osteogenesis is evident from Von Kossa staining for mineralization (black); and chondrogenesis is evident from toluidine blue staining type II collagen (purple). Arrows: differentiation area. Scale bar, 50 μm (**b**,**c**). Experiments (**b**,**c**) were performed for two obese patients with similar results.

We previously developed an approach to enumerate ASCs as CD34brightCD45−CD31− cells by multi-parametric flow cytometric analysis of peripheral blood mononuclear cells (PBMCs)^31^,^40^. We applied this methodology to assess stromal cell mobilization into the systemic circulation of randomly selected 24 non-obese (BMI<30) and 21 obese (BMI>30) patients (Supplementary Fig. 1a). As internal controls, we enumerated haematopoietic and endothelial-circulating progenitors (CPC) as CD34brightCD31dimCD45dim cells, CD34dimCD31brightCD45−CD31− mature endothelial cells and CD34brightCD45+CD31− leukocytes (Supplementary Fig. 1b). To set baseline circulation levels, PBMCs of 12 non-obese cancer-free donors^31^ were used. For prostate cancer patients, we detected a significant increase in circulation of cells with the ASC and CPC immunophenotypes. Interestingly, ASC (but not CPC) circulation was significantly higher for obese prostate cancer patients (Fig. 1a).

To confirm the flow cytometry data, we analysed plastic-adherent cells isolated from the peripheral blood. Cells with the ASC morphology reported previously^31^,^40^ were isolated for six out of six obese prostate cancer patients, while they could not be recovered from control donors (Fig. 1b). On expansion in culture, stromal cells from obese patient blood robustly differentiated into adipocytes, osteoblasts and chondrocytes, confirming them as mesenchymal progenitors (Fig. 1c). The observed expression of CD34, a marker not expressed by bone marrow MSCs^41^, suggests that MSCs circulating in obesity are ASCs mobilized from WAT.

### Obesity-associated CXCL1 expression in human prostate cancer

**Figure 2:**
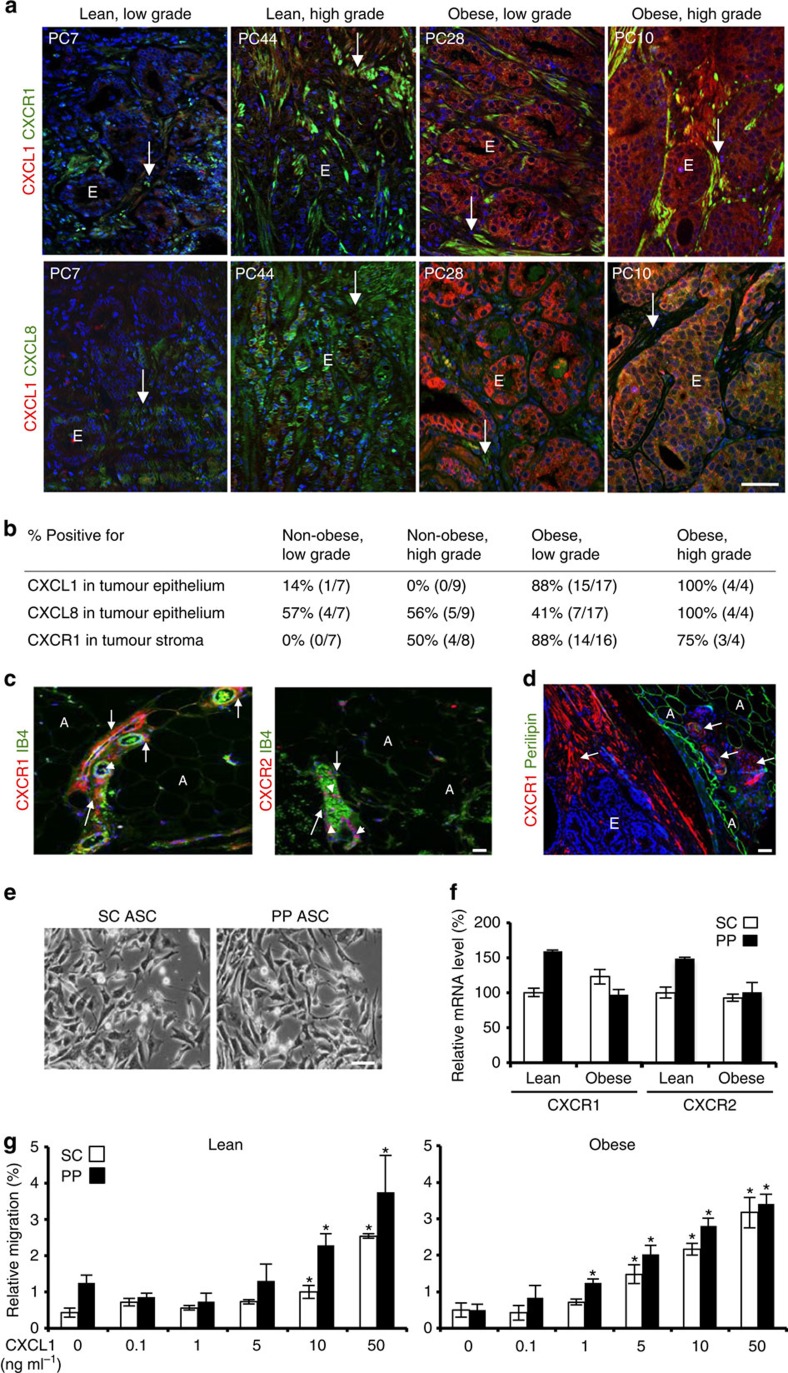
Human chemokine/receptor expression in prostate cancer and WAT. (**a**) Immunofluorescence (IF) analysis of representative tumour samples from identified patients belonging to the specified groups with antibodies against CXCL1, CXCL8 and CXCR1 with red and green fluorophore-conjugated secondary antibodies. Yellow colour indicates protein co-expression. Arrows, stroma; E, epithelium. Blue, nuclei. Scale bar, 100 μm. (**b**) Analyses of protein expression in the tumour based on the data for 37 patients (Supplementary Fig. 1c). Per cent of patients presenting indicated protein expression within each group is shown. (**c**) IF analysis of representative human periprostatic WAT samples with antibodies against CXCR1 and CXCR2 counterstained with endothelium-specific isolectin B4 (IB4), showing that perivascular stroma (arrows) expresses CXCR1, while CXCR2 is only expressed in leukocytes (arrowheads) observed inside blood vessels. A, adipocytes. Scale bar, 100 μm. (**d**) IF analysis of a representative human tumour/PP WAT junction showing CXCR1 expression by the stroma of tumour and WAT (arrows) and perilipin-1 expression by adipocytes. Scale bar, 100 μm. (**e**) Bright-field micrographs of adherent ASCs from SC and PP WAT of an obese prostate cancer patient. Scale bar, 100 μm. (**f**) RT–PCR analysis quantifying CXCR1 and CXCR2 messenger RNA expression in ASCs isolated from SC and PP WAT of obese and non-obese patients. Data are normalized to 18S RNA. (**g**) ASCs isolated from SC and PP WAT of obese and non-obese patients were subjected to migration through 8-μm pores towards increased indicated concentrations of CXCL1 in a transwell chamber. Plotted are relative migrated cell numbers normalized to migration of SC ASCs towards 10% FBS (set at 100%). Experiments in (**a**,**c**–**e**) were performed for three patients; representative images are shown. In **f** and **g**, graphs show mean±s.e.m. for technical triplicates; *P<0.05 (Student's *t*-test) versus control (no CXCL1).

To gain insight into the molecular pathways regulating trafficking of ASCs to tumours, we chose as candidates CXCL1 and CXCL8, the two chemokines secreted by human cancer cells, whose expression has previously been associated with prostate cancer aggressiveness^39^,^42^,^43^. We performed immunofluorescence analysis of CXCL1 and CXCL8 in radical prostatectomy specimens from 16 non-obese (lean and overweight) and 21 obese patients at our institution (Supplementary Fig. 1c). Representative data for obese and non-obese patients with low-grade (Gleason score ≤3+4) and high-grade (Gleason score ≥4+3) prostate cancer are shown in Fig. 2a and Supplementary Fig. 2, and summarized in Supplementary Fig. 1c. In lean patients, no CXCL1 or CXCL8 protein expression was detected in non-malignant prostate epithelium (Supplementary Fig. 2a). CXCL8 was detectable in malignant epithelium in approximately half of low-grade tumours analysed irrespective of BMI. In high-grade tumours, CXCL8 expression in malignant epithelium was also observed for five out of nine non-obese patients and for all obese patients (Fig. 2a,b; Supplementary Fig. 2c,d). This indicates that CXCL8 expression is associated with malignant transformation (Fig. 2b). CXCL1 expression pattern was strikingly different (Supplementary Fig. 1c). CXCL1 was detectable in non-malignant prostate epithelium for 40% of obese patients (Supplementary Fig. 2b). In malignant prostate epithelium, a high level of CXCL1 expression was observed for 19 out of 21 obese patients, irrespective of tumour grade (Fig. 2a,b; Supplementary Fig. 1c). In contrast, CXCL1 was detectable in malignant prostate cells of only 1 out of 16 non-obese patients (Fig. 2a,b; Supplementary Fig. 2c,d). Our data indicate that CXCL1 expression in non-malignant prostate epithelium is obesity dependent and is further induced in malignant cells of obese patients. We also performed immunofluorescence on a Biomax tissue microarray HPro-Ade96Sur-01s containing 36 prostate adenocarcinoma cores with matched normal adjacent tissue. This analysis revealed that CXCL1 expression was high in malignant epithelium of three out of three patients who subsequently died from cancer, while it was variable in other cases and absent in normal prostate controls (Supplementary Fig. 2e).

### Human ASCs and tumour stromal cells express CXCR1

To assess whether ASCs could be recruited in response to CXCL1 secreted by tumours in obesity, we performed immunofluorescence analysis of CXCR1 and CXCR2, the receptors of CXCL1 and CXCL8. In both obese and non-obese patients, we observed clear CXCR1 expression on perivascular cells (ASCs) in both SC and PP WAT, as well as on intravascular leukocytes (Fig. 2c,d). In contrast, CXCR2 expression was restricted to circulating leukocytes observed within the lumen of cross-sectioned blood vessels in all WAT samples analysed (Fig. 2c). These data suggested that CXCR1, rather than CXCR2, is likely to be the physiologically relevant ASC receptor for tumour-secreted CXCL1 and CXCL8.

Immunofluorescence analysis of tissues from prostate cancer patients demonstrated the presence of cells with strong CXCR1 expression in the tumour stroma of some, but not all patients (Fig. 2a; Supplementary Fig. 2b–e). The data on CXCR1-bright cell infiltration in obese and lean patients are summarized in Supplementary Fig. 1c. The presence of CXCR1-bright cells in the prostate stroma was observed for 50% of non-obese and for 75% of obese patients with high-grade cancer (Fig. 2a,b). Interestingly, cells strongly positive for CXCR1 were not observed in the tumour stroma of non-obese patients with low-grade cancer. In contrast, 88% of obese patients with low-grade cancer had a high presence of CXCR1-bright cells in the prostate stroma (Fig. 2a,b). Like in WAT, CXCR2 expression on stromal cells was not detected (Supplementary Fig. 2f). These data indicate that expression of CXCL1 by prostate epithelium in obesity correlates with prostate stroma infiltration by cells expressing its receptor CXCR1. Analysis of 36 prostate samples in a tissue microarray revealed that CXCR1-bright stroma was present in tumours of three out of three patients who subsequently died from cancer, while it was variable in other cases and absent in normal prostate controls (Supplementary Fig. 2e). Our results suggest that the obesity-associated CXCL1 expression by prostate epithelium could be responsible for recruitment of ASCs, the WAT stromal cells expressing CXCR1.

### Human ASCs migrate towards CXCL1 and CXCL8

To test whether ASC migration can be activated by chemokines analysed, we isolated ASCs from PP and SC WAT of non-obese and obese patients. All patient-derived ASC cultures displayed typical ASC morphology in culture (Fig. 2e). Interestingly, after several passages, ASCs expressed both CXCR1 and CXCR2 (Fig. 2f). Using a transwell assay, we tested directional migration of ASCs plated into the upper chamber towards chemokines added to the lower chamber across the 8-μm pore membrane. Both PP and SC ASCs from non-obese and obese patients displayed dose-dependent migration towards CXCL1 (Fig. 2g). Notably, ASCs from PP WAT of non-obese patients, which were found to express more CXCR1 and CXCR2 than ASCs from SC WAT (Fig. 2f), displayed a trend for higher migration towards CXCL1 (Fig. 2g). Human ASCs also migrated towards CXCL8 (Supplementary Fig. 3a). Reparixin, a small-molecule inhibitor of CXCR1 and CXCR2 (ref. 44) reduced human ASC migration towards CXCL1 and CXCL8 (Supplementary Fig. 3a). To compare ASC response with that of monocytes, known to be guided by this chemokine gradient^44^, conditionally immortalized macrophages derived from ‘immorto-mice' as described previously^29^ were used. Human ASCs and macrophages displayed a comparable capacity to migrate towards either CXCL1 or CXCL8 in serum-free medium (Supplementary Fig. 3b). Importantly, ASC chemotaxis towards either CXCL1 or CXCL8 was inhibited with both CXCR1-blocking and CXCR2-blocking antibodies (Supplementary Fig. 3b).

In contrast to ASCs, we found that human bone-marrow-derived MSCs lack CXCR1 expression (Supplementary Fig. 4a) and their migration was stimulated by CXCL1 to a much lower extent (Supplementary Fig. 4b). These data indicate that ASCs, but not bone marrow MSCs, are chemoattracted to the tumour-secreted CXCR1/2 ligands.

### The CXCL1–CXCR1/2 gradient is conserved in mice

While mice do not have the CXCL8 orthologue, they have CXCL1, as well as orthologues of both human receptors, CXCR1 and CXCR2 (refs 45, 46). To test whether the mouse model is appropriate to study the CXCL1 gradient in obesity/cancer, we analysed the systemic circulation of 50 mouse cytokines and chemokines represented in the Luminex panel. C57BL/6 mice raised on chow or high-fat diet were grafted with cancer cells, and plasma samples were analysed in a multiplex assay for non-obese and obese mice bearing tumours. This unbiased approach identified CXCL1 as the only chemokine in the panel that circulated at more than a twofold higher level in obese animals, compared with non-obese animals bearing tumours (Supplementary Fig. 5a).

**Figure 3:**
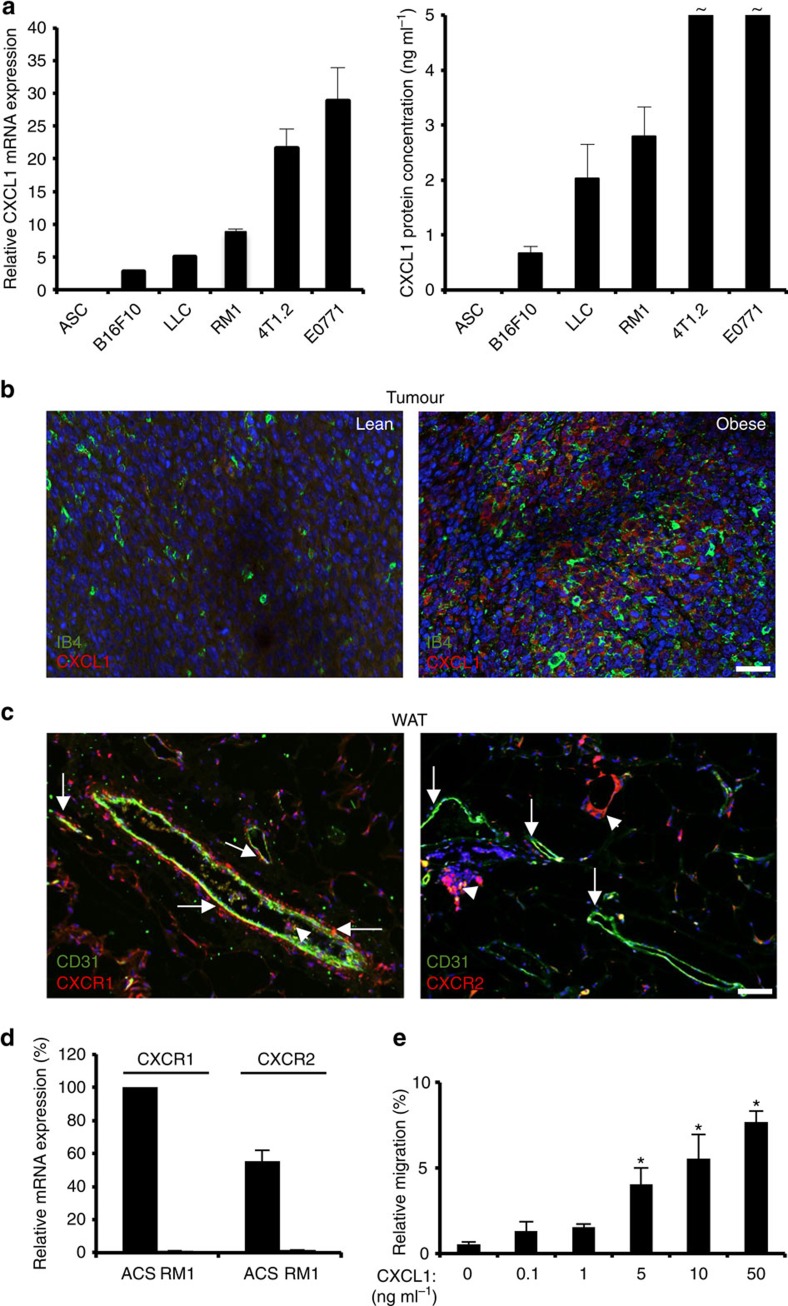
CXCL1 secreted by mouse tumour cells attracts CXCR1-expressing ASCs. (**a**) Left: RT–PCR analysis of CXCL1 messenger RNA expression in mouse ASCs and indicated mouse tumour cell lines. Data were normalized to 18S RNA. Right: CXCL1 protein concentration in medium conditioned by mouse ASCs and indicated tumour cell lines measured by ELISA. ∼ values above 5 ng ml^−1^. (**b**) Immunofluorescence (IF) analysis of tumours grown in non-obese and obese mice with anti-CXCL1 antibodies indicating CXCL1 expression increased in obesity. Endothelium is counterstained with isolectin B4 (IB4). Nuclei are blue. Scale bar, 100 μm. (**c**) IF analysis of mouse WAT with antibodies against CXCR1 (left) and CXCR2 (right), and endothelial cell marker CD31. Note CXCR1 expression in perivascular ASCs (arrows), while CXCR2 is expressed only in circulating leukocytes and macrophage crown structures (arrowheads). Scale bar, 100 μm. (**d**) RT–PCR analysis of CXCR1 and CXCR2 expression in mouse ASCs and RM1 tumour cells. Data were normalized to 18S RNA. (**e**) Primary mouse ASCs were subjected to migration through 8-μm pores towards indicated concentrations of CXCL1 in a transwell chamber. In **a**,**d** and **e**, graphs show mean±s.e.m. for technical triplicates; *P<0.05 versus control (Student's *t*-test). Experiments in **b** and **c** were performed for three mice with similar results; representative images are shown.

To use the mouse model for further investigation of CXCL1 function in prostate cancer, we screened a number of mouse tumour cell lines for CXCL1 expression. Mouse ASCs were used as a negative control. CXCL1 messenger RNA expression was found to be high for E0771 and 4T1.2 breast adenocarcinoma, as well as Lewis lung carcinoma previously shown to attract ASCs^27^,^29^. RM1, an androgen receptor-positive/androgen-independent prostate adenocarcinoma cell line^47^, is highly expressed in CXCL1 (Fig. 3a). Secretion of CXCL1 protein by RM1 cells was confirmed by enzyme-linked immunosorbent assay (ELISA) (Fig. 3a). We then grafted C57BL/6 mice raised on low-fat diet (LFD) or high-fat diet (HFD) with RM1 cells SC and analysed tumours from non-obese and obese mice. Immunofluorescence analysis demonstrated the CXCL1 expression by tumour cells, which was notable higher in obese mice (Fig. 3b). On the basis of these observations, we chose RM1 grafts as a mouse model of aggressive prostate cancer for subsequent studies.

Using tissues from the same mice, we assessed the expression of CXCR1 and CXCR2 murine orthologues in WAT by immunofluorescence. Our analysis revealed expression of CXCR1 by perivascular ASCs, as well as by leukocytes infiltrating WAT (Fig. 3c). As in humans, CXCR2 was detected in intravascular leukocytes and the crown-like structures formed by macrophages, which were abundant in WAT of obese animals, while ASCs are CXCR2 negative (Fig. 3c). Like for human ASCs (Fig. 2f), expression of both CXCR1 and CXCR2 was observed for cultured mouse ASCs (Fig. 3d). We demonstrated that, like human ASCs, in the transwell assay, primary mouse ASCs migrate towards CXCL1 in a dose-dependent manner (Fig. 3e).

### ASCs migrate towards chemokines secreted by tumour cells

**Figure 4:**
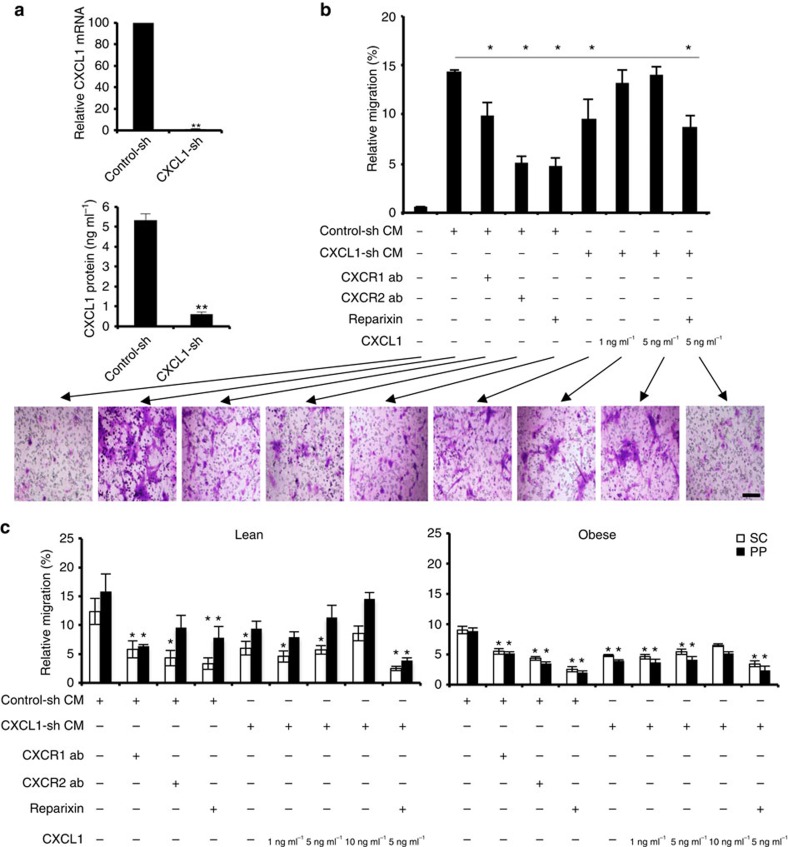
CXCL1 and CXCR1/2 signalling blockade inhibits ASC migration. (**a**) Upper: quantitative RT–PCR analysis of CXCL1 messenger RNA (mRNA) expression in RM1 cells transduced with control shRNA (control-sh) or with shRNA silencing CXCL1 (CXCL1-sh), normalized to 18S RNA. Lower: CXCL1 protein concentration in medium conditioned by control-sh- or CXCL1-sh-transduced RM1 cells measured by ELISA. **P<0.01 (Student's *t*-test). (**b**) Mouse ASCs were subjected to migration through 8-μm pores to a transwell chamber with conditioned medium (CM) from control-sh- or CXCL1-sh-RM1 cells. CM was supplemented with indicated concentrations of CXCL1, antibodies (abs) blocking CXCR1 or CXCR2, or CXCR1/CXCR2 inhibitor, reparixin, where indicated (+). Shown below are representative bright-field micrographs of crystal violet-stained transwell membranes corresponding to each treatment group. Scale bar, 100 μm. (**c**) ASCs isolated from SC and PP WAT of an obese and a non-obese prostate cancer patient were subjected to migration through 8-μm pores to a transwell chamber with CM from control-sh- or CXCL1-sh-RM1 cells. CM was supplemented with indicated concentrations of CXCL1, ab blocking CXCR1 or CXCR2, or reparixin where indicated (+). In **b** and **c**, plotted are relative migrated cell numbers normalized to migration of ASCs towards 10% FBS (set at 100%). For all panels, experiments were performed in technical triplicate. Graphs show mean±s.e.m. *P<0.05 versus control-sh CM (Student's *t*-test).

Next, we performed experiments to confirm that CXCL1 secreted by cancer cells has a chemotactic activity on ASCs. Using lentivirus-expressing short hairpin RNA (shRNA), we silenced CXCL1 expression in RM1 cells and analysed these cells in parallel with RM1 cells expressing control (untargeted) shRNA. Silencing of sh-CXCL1 in RM1 cells was confirmed by quantitative RT–PCR and ELISA on cell-conditioned medium (Fig. 4a). In the transwell assay, control-sh-RM1 cell-conditioned medium had a strong chemotactic effect on mouse ASCs, which was significantly reduced by CXCL1 silencing (Fig. 4b). This reduction in ASC migration was restored by increasing doses of CXCL1 added to the CXCL1-sh-RM1 cell-conditioned medium (Fig. 4b). Reparixin decreased migration of ASCs towards control-sh-RM1 cell-conditioned medium or towards CXCL1-sh-RM1 cell-conditioned medium supplemented with CXCL1 (Fig. 4b). Inhibitory antibodies against either CXCR1 or CXCR2 also interfered with migration of ASCs towards control-sh-RM1 cell-conditioned medium (Fig. 4b). This is consistent with our observation that in cell culture both CXCR1 and CXCR2 are expressed in ASCs (Figs 2f and 3d).

We confirmed that human ASCs also respond to CXCL1 from tumour cell-conditioned medium in the transwell assay. ASCs from either SC or PP WAT of both non-obese and obese patients migrated towards control-sh-RM1 cell-conditioned medium better than towards CXCL1-sh-RM1 cell-conditioned medium (Fig. 4c). The reduced migration towards CXCL1-sh-RM1 cell-conditioned medium was at least partly restored by CXCL1 supplementation. ASC migration towards control-sh-RM1 cell-conditioned medium was partly inhibited by reparixin, as well as by blocking anti-CXCR1 and anti-CXCR2 antibodies (Fig. 4c). These trends were observed for both SC and PP ASCs.

To compare human ASC response with CXCL1 and CXCL8, we used conditioned medium from human DU145 adenocarcinoma cells that secrete both chemokines (Supplementary Fig. 3c). Migration of human ASCs and of primary PBMC-derived monocytes, as well as of immortalized mouse macrophages, towards prostate cancer cell-secreted chemokines was analysed. Blockade of either CXCL1 or CXCL8 significantly reduced chemotaxis of each cell type to a similar degree (Supplementary Fig. 3d). On blockade of both chemokines, ASC migration was additively decreased to the level observed in the presence of either CXCR1-inhibiting or CXCR2-inhibiting antibody (Supplementary Fig. 3d). Combined, these results indicate that both mouse and human ASCs are chemoattracted to CXCL1, as well as to CXCL8 secreted by cancer cells.

### CXCR1/CXCR2 signalling mediates ASC trafficking to tumours

**Figure 5:**
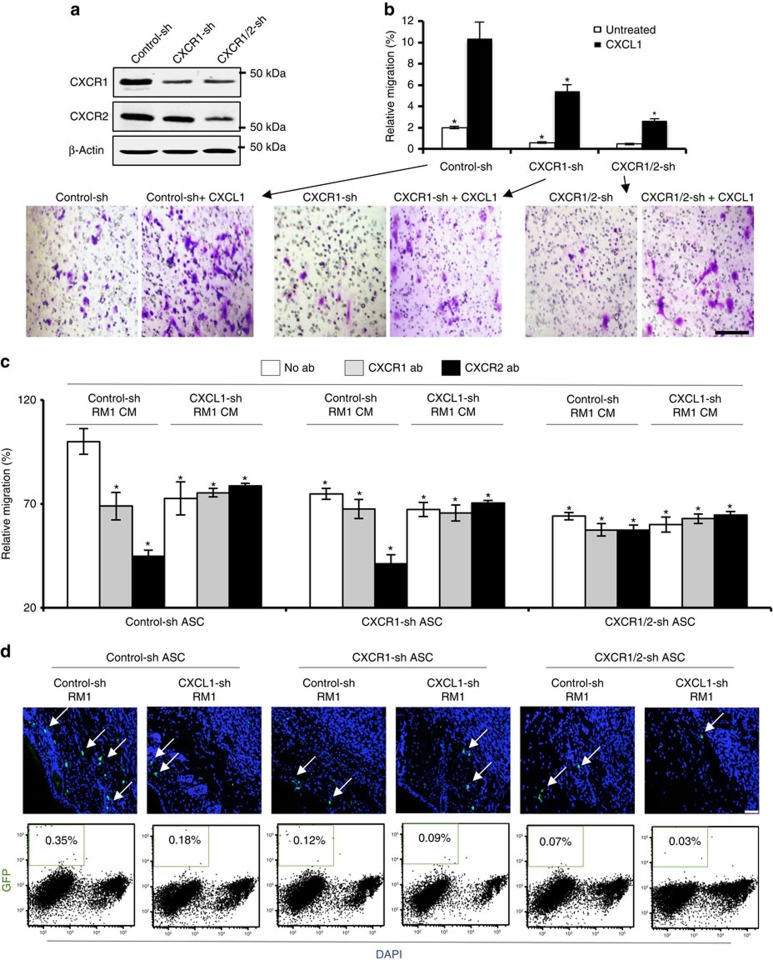
CXCR1/CXCR2 silencing inhibits ASC migration to tumours. (**a**) Immortalized GFP-expressing mouse ASCs transduced with an untargeted shRNA (control-sh), or shRNA targeting either only CXCR1 (CXCR1-sh) or both CXCR1 and CXCR2 (CXCR1/2-sh) were subjected to immunoblotting with CXCR1 and CXCR2 antibodies. Experiment was performed twice with similar results; representative scans are shown. (**b**) Cells from **a** were subjected to transwell chamber migration through 8-μm pores toward medium with or without 5 ng ml^−1^ CXCL1. Plotted are relative migrated cell numbers normalized to migration of SC ASCs towards 10% FBS (100%). Shown below are representative bright-field micrographs of crystal violet-stained transwell membranes corresponding to each treatment group. Scale bar, 100 μm. (**c**) Cells from **a** were subjected to transwell chamber migration through 8-μm pores towards CM from control-shRNA or CXCL1-shRNA RM1 cells supplemented with antibodies (abs) against CXCR1 or CXCR2 where indicated. (**d**) Representative sections of tumours formed by control-sh-RM1 and CXCL1-sh RM1 cells grafted into the nude mice (*n*=6) that received subcutaneous contra-lateral injections of control-sh, CXCR1-sh or CXCR1/2-sh GFP-expressing ASCs. Arrows indicate tumour-engrafted ASCs (green), recruitment of which is reduced by both CXCL1 and CXCR1 silencing. Scale bar, 100 μm. Nuclei are blue. Representative flow cytometric analyses of suspended tumour cells enumerating injected ASCs (GFP+) among viable (4,6-diamidino-2-phenylindole (DAPI) negative) cells. Note ASC frequency reduction by CXCL1 and CXCR1 silencing. For **b**–**d**, experiments were performed in technical triplicate. Graphs show mean±s.e.m.; *P<0.05 (Student's *t*-test) versus control-sh CM.

We next tested whether the CXCL1 receptors expressed in ASCs play a role in their migration to tumours. Mouse ASCs conditionally immortalized as described previously^29^ were transduced with lentivirus-expressing shRNA targeting either only CXCR1 or both CXCR1 and CXCR2 through a sequence conserved in the two receptors (Supplementary Fig. 5b). The selectivity of these knockdown constructs was confirmed by measuring CXCR1 and CXCR2 expression (Fig. 5a; Supplementary Fig. 5c). ASCs transduced with control shRNA displayed migration towards CXCL1 in the transwell assay, confirming the receptor activity in this cell model (Fig. 5b). This CXCL1-directed chemotaxis was reduced on silencing of CXCR1 and further reduced on combined CXCR1/CXCR2 silencing, although consistent with residual CXCR1 and CXCR2 expression remaining (Fig. 5a), CXCL1 was still capable to partly activate migration of cells transduced with the knockdown constructs (Fig. 5b).

Next, we used the transwell assay to confirm that CXCL1 secreted by tumours chemoattracts the immortalized ASCs. Control ASCs transduced with untargeted shRNA migrated towards control-sh-RM1 cell-conditioned medium significantly better than towards CXCL1-sh-RM1 cell-conditioned medium (Fig. 5c). As expected, migration of control ASCs towards control-sh-RM1 cell-conditioned medium, but not towards control-sh-RM1-conditioned medium, was inhibited by anti-CXCR1 and anti-CXCR2 antibodies (Fig. 5c). ASCs with silenced CXCR1 and CXCR1/CXCR2 displayed a significantly reduced migration towards control-sh-RM1 cell-conditioned medium (Fig. 5c). As expected, CXCR1-sh ASC migration towards control–sh-RM1 cell-conditioned medium was further reduced by anti-CXCR2 antibody, but not by anti-CXCR1 antibody (Fig. 5c). Neither anti-CXCR1 nor anti-CXCR2 antibody reduced migration of CXCR1/2-sh ASCs towards control-sh-RM1 cell-conditioned medium below baseline migration of these cells towards CXCL1-sh-RM1 cell-conditioned medium (Fig. 5c), consistent with the targeting of both receptors in these cells. The capacity of CXCL1 to activate CXCR1 was confirmed for human ASCs. On CXCR1 blockade, CXCL1 capacity to chemoattract ASCs was decreased to the same extent as on CXCR2 blockade, and chemotaxis was completely abrogated only on combined blockade of CXCR1 and CXCR2 (and to CXCL8; Supplementary Fig. 3b).

We also tested whether CXCL1–CXCR1/CXCR2 chemotaxis mediates ASC trafficking *in vivo*. To avoid immunorejection of immortalized ASCs derived from mice with a mixed genetic background^29^, we used an immunodeficient (nude) mouse model. Mice were SC grafted with control-sh-RM1 or CXCL1-sh-RM1 cells and were allowed to form tumours. The immortalized ASCs (Fig. 5a), which express the green fluorescent protein (GFP) and thereby can be traced in tissues, were injected SC into the contra-lateral side. Analysis of tissue sections revealed the recruitment of GFP+ ASCs by tumours (Fig. 5d). Using flow cytometry, we quantified that the frequency of GFP+ ASCs was reduced threefold on CXCR1 silencing and fivefold on combined CXCR1/CXCR2 silencing (Fig. 5d). Recruitment to CXCL1-sh-RM1 tumours was also reduced by 2-fold for control ASCs and by 10-fold for CXCR1/CXCR2 knockdown ASCs (Fig. 5d). Combined, these data indicate that ASCs are attracted by tumour-secreted CXCL1 synergistically acting through CXCR1 and CXCR2.

### CXCL1-dependent recruitment of ASCs to tumours in obesity

**Figure 6:**
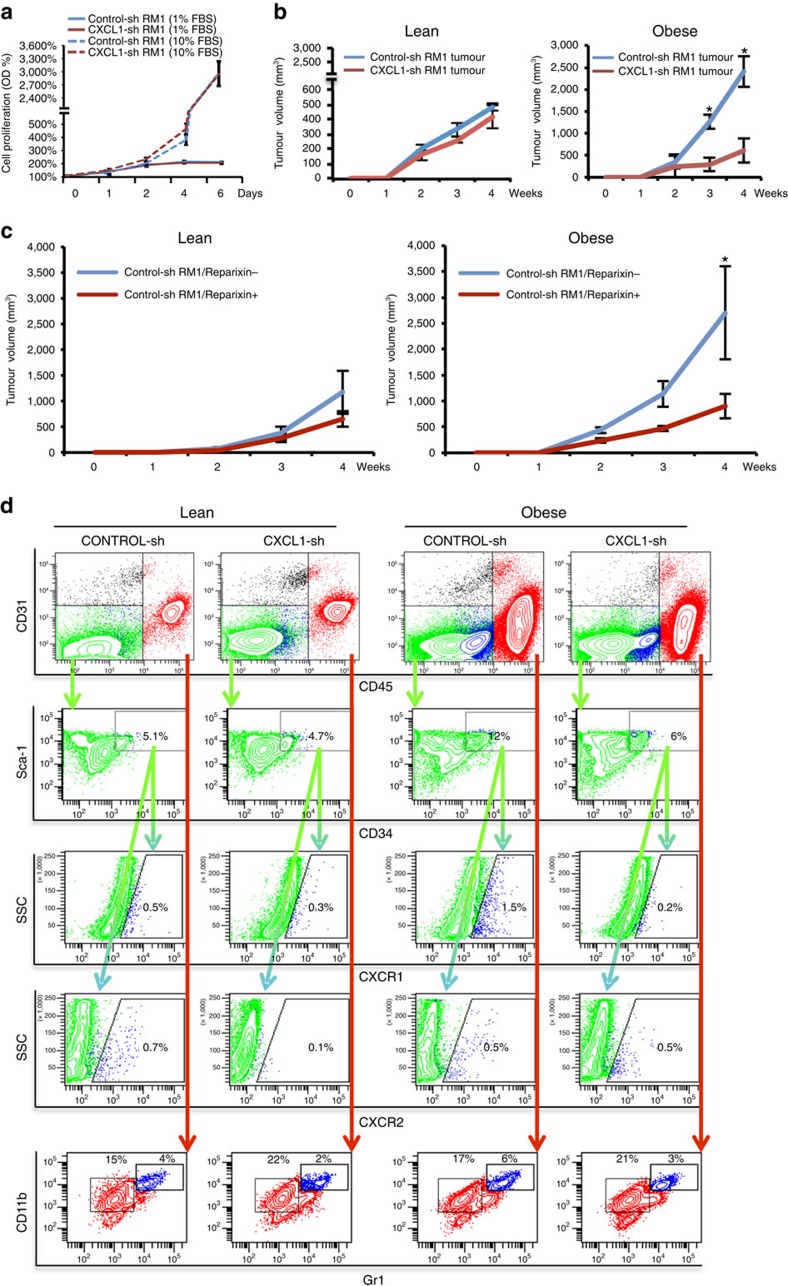
CXCL1 silencing inhibits ASC trafficking to tumours and tumour growth. (**a**) Proliferation of control-shRNA and CXCL1-shRNA RM1 cells growing in 1or 10% FBS assessed by MTT assay. Plotted is relative fluorescence unit (RFE) increase above day 0 value from technical triplicates. (**b**) RM1 cells transduced with an untargeted shRNA (control-sh) or shRNA-targeting CXCL1 (CXCL1-sh) were subcutaneously grafted into non-obese or obese mice (*n*=5 per group), and tumour volume was measured weekly. (**c**) Growth of RM1 grafts in mice treated or not treated with reparixin (*n*=5 per group) in lean and obese mice. In **a**–**c**, graphs show mean±s.e.m.; *P<0.05 (Student's *t*-test). (**d**) Representative flow cytometric gating to separate tumour CD45− (stromal, vascular and malignant) and CD45+ (haematopoietic) cells. Gating of CD45−CD34+Sca1+ ASCs based on side scatter (SSC) and CXCR1/2 expression reveals increased frequency of CXCR1+ ASCs in control-shRNA, but not in CXCL1-shRNA tumours in obese mice. Gating of CD45− leukocytes based on CD11b and Gr1 expression reveals reduced frequency of CD11b+Gr1+ MDSCs in CXCL1-shRNA tumours in both lean and obese mice. % of total viable cells is shown. Analyses were performed twice with similar results.

To investigate the relevance of CXCL1-dependent ASC trafficking to cancer progression, we first demonstrated that proliferation of CXCL1-sh-RM1 and control-sh-RM1 cells was non-distinguishable at both high serum and low serum concentrations (Fig. 6a). There was also no difference in the growth rate of tumours formed by SC-grafted CXCL1-sh-RM1 and control-sh-RM1 cells in lean mice (Fig. 6b). In obese mice, growth of control-sh-RM1 tumours was significantly increased consistent with the previous reports^27^. This increase was not observed for CXCL1-sh-RM1 tumours, indicating the importance of CXCL1 for the obesity-mediated tumour growth promotion (Fig. 6b). Consistent with these data, growth of RM1 grafts was significantly inhibited by reparixin in obese, but not in lean mice (Fig. 6c). Together, these results indicate that CXCL1 signalling via CXCR1 and CXCR2 mediates the obesity-associated stimulation of tumour growth.

To determine whether reduced ASC recruitment explains the lack of obesity effect on the CXCL1-silenced tumours, we performed flow cytometric analysis of tumour cell suspension. As expected, CXCR1+ and CXCR2+ cells were observed among both CD45+ (haematopoietic) and CD45− (mixed malignant, stromal and vascular) cells (Fig. 6d). On the basis of CD34+CD31−CD45−CD34+Sca1+ immunophenotype reported for ASCs previously^48^, we set the gate for their enumeration. Consistent with the tumour growth data, in obese, but not in lean, mice there was a sevenfold higher frequency of CXCR1+ ASCs observed in CXCL1-sh-RM1 tumours, compared with control-sh-RM1 tumours (Fig. 6d). In contrast, there was no difference in the frequency of CXCR2+ cells between control-sh and CXCL1-sh RM1 tumours. These data indicate that CXCL1 is required for the recruitment of CXCR1-expressing ASCs to tumours associated with obesity. We also enumerated CD45+ leukocytes to determine whether they could contribute to obesity-associated tumour growth promotion. Because certain myeloid cells are known to be chemoattracted by CXCL1 and promote tumour growth^44^, we analysed their frequency based on markers CD11b and Gr1. As expected, the frequency of CD11b+Gr1+ MDSCs, a population including neutrophils, was decreased by twofold in tumours on CXCL1 silencing in both lean and obese mice (Fig. 6d).

**Figure 7:**
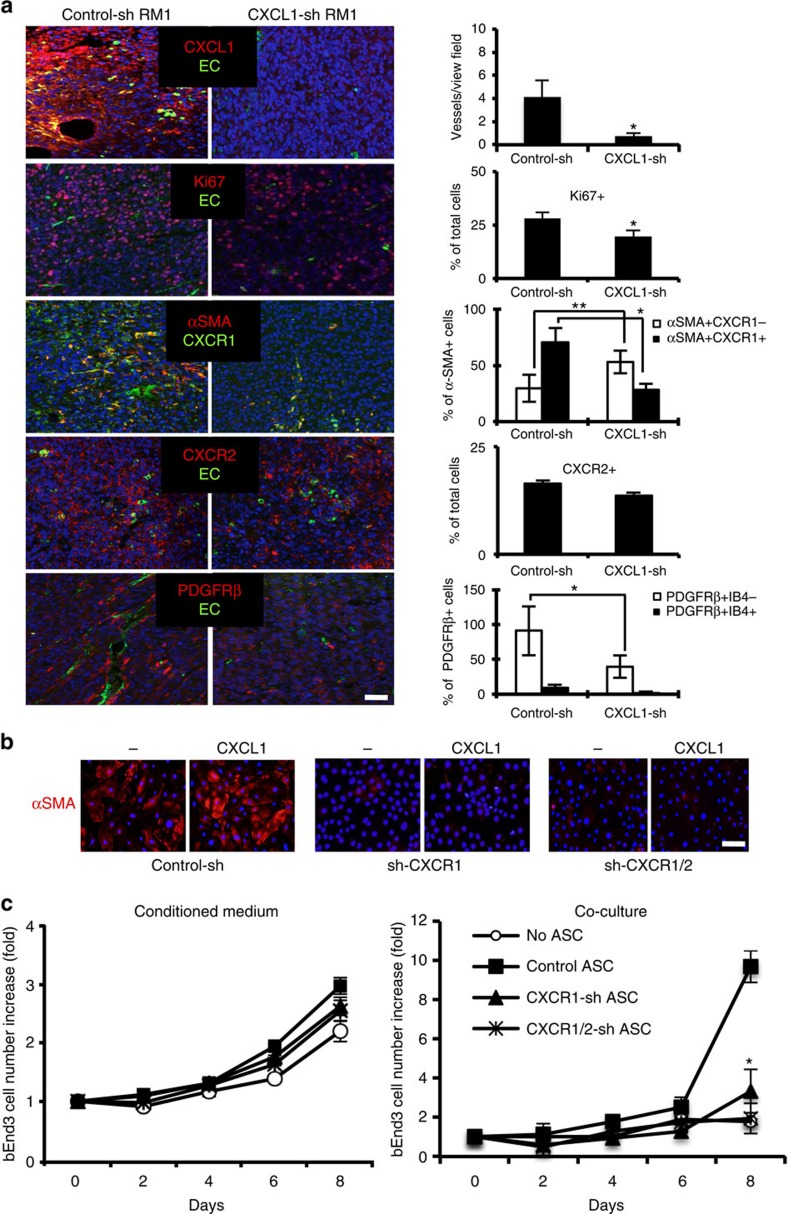
CXCL1 function in ASCs αSMA expression and endothelium stimulation. (**a**) Representative immunofluorescence analysis of tumours grown by RM1 cells transduced with an untargeted shRNA (control-sh) or shRNA-targeting CXCL1 (CXCL1-sh) in obese mice with antibodies against CXCL1, CXCR1, CXCR2, αSMA, PDGFRβ and Ki67. Endothelial cells (ECs) are counterstained with isolectin B4 (IB4). Scale bar, 100 μm. Nuclei are blue. Graphs show data mean±s.e.m.; *P<0.05 versus control-sh (Student's *t*-test). Quantification based on 10 view fields (10 × ) shows lower frequency of vessels and of CXCR1+/αSMA+, PDGFRβ+ and Ki67+ cells in CXCL1-sh tumours. (**b**) αSMA immunofluorescence (red) analysis of immortalized mouse ASCs transduced with control-sh, CXCR1-sh or CXCR1/2-sh. Medium contained 5 ng ml^−1^ CXCL1 where indicated. Scale bar, 100 μm. Nuclei are blue. Data representative of 10 images analysed are shown. (**c**) Proliferation of bEnd.3 cells in direct co-culture with GFP-labelled immortalized mouse ASCs transduced with control-sh, CXCR1-sh or CXCR1/2-sh, or incubated with conditioned medium from these ASCs. Plotted are numbers of bEnd.3 (FITC−) cells collected after the indicated numbers of days. Graphs show mean±s.e.m. for technical triplicates; *P<0.05; **P<0.01 versus control-sh (Student's *t*-test).

We also used immunofluorescence analysis to validate the flow cytometry data for obese animals. CXCL1 expression observed in control-sh-RM1 tumours was expectedly absent in CXCL1-sh-RM1 tumours, indicating cancer cells as the main source of tumour-secreted CXCL1 (Fig. 7a). CXCR1+ cells were abundant in the stroma of control-sh-RM1 tumours, while in CXCL1-sh-RM1 tumours they were sparse (Fig. 7a). In contrast, the frequency of CXCR2+ cells was comparable for control-sh-RM1 and CXCL1-sh-RM1 tumours (Fig. 7a). These results indicate that CXCL1-mediated recruitment of CXCR1+ stromal cells accounts for obesity-associated tumour growth promotion.

### CXCR1-mediated angiogenic αSMA induction in ASCs

Finally, we assessed the mechanisms through which ASCs recruited to tumours promote tumour growth in obesity. Ki67 immunofluorescence of tumours from obese mice demonstrated a higher frequency of cell proliferation in control-sh-RM1 tumours compared with CXCL1-sh-RM1 tumours (Fig. 7a). Proliferation typically was observed in vascularized tumour areas, which were less abundant in CXCL1-sh-RM1 tumours. Compared with control-sh-RM1 tumours, blood vessels in CXCL1-sh-RM1 tumours were thinner and less wide open (Fig. 7a). Perivascular cells expressing PDGFRβ, a mesenchymal marker of ASC^40^,^49^, were significantly less abundant in CXCL1-sh-RM1 tumours compared with control-sh-RM1 tumours (Fig. 7a).

Expression of αSMA, a myofibroblast marker^13^, is higher in CAFs derived from ASCs, as compared with CAFs derived from the bone marrow^20^. Consistent with that notion, αSMA+ cells were abundant in control-sh-RM1 tumours, but not in CXCL1-sh-RM1 tumours grown in obese animals (Fig. 7a). Confirming αSMA+ stromal cells being largely of ASC origin, in control tumours, the majority of αSMA+ cells were found to also express CXCR1+, while this coincidence of expression was significantly reduced in CXCL1-sh-RM1 tumours (Fig. 7a). Interestingly, αSMA expression was induced on CXCL1 stimulation in immortalized ASCs grown in culture (Fig. 7b). Moreover, CXCR1-sh and CXCR1/CXCR2-sh ASCs completely lacked αSMA, and αSMA expression was not CXCL1-inducible on receptor knockdown (Fig. 7b). This indicated that the CXCL1–CXCR1/CXCR2 signalling regulates αSMA induction in ASCs, which might predetermine acquisition of their tumour-promoting properties.

To test whether αSMA+ ASCs directly stimulate malignant cells, we tested the effect of ASC-conditioned medium on RM1 cell growth. RM1 cell proliferation was elevated in the presence of medium from cultures of ASCs derived from both PP and SC WAT of non-obese and obese patients. Because this effect was weak (Supplementary Fig. 6a), we tested whether ASCs may be stimulating cancer cells in a contact-dependent manner. To assess that, proliferation of RM1 cells mixed with GFP-labelled immortalized mouse ASCs was analysed over a period of 8 days by flow cytometry. Silencing of CXCR1 and CXCR2 did not affect ASC-dependent stimulation of RM1 proliferation (Supplementary Fig. 6b,c). These data indicate that ASCs do not have a direct CXCL1 signalling-dependent effect on cancer cells.

On the basis of the reported pro-angiogenic function of ASCs^40^, we hypothesized that CXCL1 signalling is a requisite for ASCs effect on the endothelium. Proliferation of bEnd.3 endothelial cells, which display ASC-dependent vasculogenic properties^50^, was only weakly promoted by ASC-conditioned medium (Fig. 7c). To test whether ASCs effect on endothelial cell is contact dependent, we analysed bEnd.3 endothelial cell in direct co-culture with GFP-labelled immortalized ASCs (Supplementary Fig. 6c). Indeed, ASCs significantly promoted bEnd.3 cell proliferation in a contact-dependent manner (Fig. 7c). ASCs in which CXCR1 and CXCR2 were silenced were incapable to promote endothelial cell proliferation; importantly, CXCR1 silencing was sufficient to abrogate their stimulatory effect (Fig. 7c). Combined, these data indicate that CXCL1 signalling through CXCR1 induces αSMA expression, which mediates the stimulatory effect of ASCs on the endothelium.

## Discussion

Increased adiposity in prostate cancer patients has been identified as a factor associated with disease aggressiveness^22^,^23^,^24^,^25^,^26^. Tumour stroma, composed of a mixture of various non-malignant cells types, has been identified as one of the drivers of cancer progression^15^,^18^,^19^,^51^,^52^. In mouse models, tumour growth is promoted by infiltrating ASCs, which identified these mesenchymal WAT-derived cells as a mechanistic link between obesity and cancer^20^,^27^,^34^,^53^. Our recent studies in endometrial and ovarian cancer suggested that CXCL1 and/or CXCL8 secreted by human cancer cells and signalling via their receptors CXCR1 and/or CXCR2 could be implicated in human ASC migration^30^. Here we provide evidence that cancer-induced CXCL8 and obesity-dependent CXCL1 gradients regulate ASC trafficking in the context of prostate cancer. Using clinical specimens and the mouse model, we demonstrate that ASCs express CXCR1, as well as CXCR2 spontaneously induced *ex vivo*. We show that CXCL1 and CXCL8 direct receptor-dependent migration of both SC and PP ASCs in *ex vivo* assays. We show that obese patients have increased CXCL1 expression in the prostate epithelium, increased systemic ASC mobilization and increased infiltration of CXCR1-expressing cells in the tumour stroma. We also use the mouse model to demonstrate that blockade of either CXCL1 or its receptors inhibits obesity-dependent ASC tumour trafficking and abrogates the obesity-associated promotion of tumour vascularization and growth. Combined, our results suggest that in patients, CXCR1 expressed in ASCs *in vivo*, and possibly CXCR2, direct ASC trafficking towards CXCL8 gradient activated in cancer and CXCL1 gradient in addition activated in obesity (Supplementary Fig. 7a).

While the role of CXCL8 has been comprehensively studied in the context of prostate cancer and other cancers^42^,^43^,^54^, CXCL1 function in cancer remains insufficiently understood. Our study links obesity-dependent CXCL1/CXCR1 gradient activation in the prostate with obesity-dependent cancer progression reported previously^22^,^23^,^24^,^25^,^26^. Analysis of the tumours of 39 patients undergoing radical prostatectomy for localized disease under our clinical protocol demonstrated that obese patients were more likely than non-obese patients to have lymph node metastases (17 versus 0%, Supplementary Fig. 1c). Furthermore, with a median follow-up of 3 years, only obese patients experienced disease recurrence (22%) or prostate cancer-specific death (*n*=1). In all three patients who progressed for whom tissue was available, strong CXCL1 expression in cancer cells was observed in all cases and infiltrating CXCR1-bright cells in two of three cases (Supplementary Fig. 1c). Given the limitations that our obese and non-obese patient cohorts were small, and follow-up duration relatively short, our data suggest that epithelial CXCL1 expression and CXCR1-positive tumour stroma in obese patients may be associated with more aggressive disease features. Further association between the CXCL1/CXCR1 gradient and patient outcome was obtained from our analysis of the tissue microarray: high epithelial CXCL1 and high stromal CXCR1 expression was observed for 100% of analysed tumours from patients who eventually deceased (Supplementary Fig. 2e). High epithelial CXCL1 and high stromal CXCR1 expression was also observed for all tumours from patients with metastasis who were still alive at the time of array assembly (data not shown). Finally, an online resource cBioPortal^55^ linked with a comprehensive profiling of 50 metastatic castration-resistant prostate cancer (CRPC) cases and 11 high-grade localized prostate cancers at the University of Michigan^56^ provides a direct evidence that prostate CXCL1 expression inversely correlates with patient survival (Supplementary Fig. 7b). Our clinical observations in prostate cancer are consistent with the previously reported^57^ correlation between increased CXCL1 tumour expression and adverse outcome in breast cancer patients. In accord with our animal data, CXCL1 circulation was found to be increased in obesity^58^. The value of CXCL1 expression as a prognostic cancer marker is to be further validated. The influence of the range of adiposity and other factors, such as age, on CXCL1 expression and CXCR1-expressing cell infiltration remains to be determined. Future studies will establish a potential synergy and the relative clinical importance of CXCL1 and CXCL8 in obesity-linked and progressive disease.

Chemokine signalling is a complex process in which cells are typically guided by multiple partly redundant chemokine gradients^35^,^36^. Ligand specificity for the CXCR1 and CXCR2 receptors has remained incompletely understood. While CXCR2 is a proven receptor for CXCL1, there are studies indicating that CXCL1 can also signal through CXCR1 (ref. 37). For example, studies on human prostate cancer cells showed that cell migration induced by CXCL1 is dependent on both CXCR1 and CXCR2 receptors^59^. The mouse CXCR1 was discovered relatively recently and only human CXCL8 was tested as its ligand^45^. Here we demonstrate that CXCL1 signals through CXCR1 and CXCR2 in both mouse and human ASCs. Although our data clearly show that ASCs are chemoattracted by CXCL1, their migration is not completely blocked by CXCR1/CXCR2 blockade, and serum has a higher capacity to induce ASC migration than CXCL1 (Figs 4b,c and 5c). These observations indicate that other pathways cooperate with CXCR1/CXCR2 signalling in guiding ASC trafficking and function. Recently, the CXCL16–CXCR6 chemokine gradient was shown to mediate MSC recruitment to prostate tumours^13^. Because the organ origin of CXCR6-positive stromal cells was not investigated, it remains to be determined whether ASC trafficking is regulated by CXCL16. It should also be noted that disruption of the CXCL1 axis blocked tumour recruitment of not only ASCs, but also MDSCs in both lean and obese mice (Fig. 6d). This was expected, as CXCL1 is known to regulate trafficking of neutrophils^44^. Because lean mice did not display a CXCL1 dependence of tumour growth, our data suggest that recruitment of ASCs, rather than of MDSCs, accounts for obesity-dependent tumour growth stimulation.

The mechanisms through which mesenchymal stroma modulates cancer progression are multifaceted^15^. Pro-angiogenic, immunomodulatory and direct tumour-trophic functions have been proposed for ASCs specifically^14^. The observed CXCL1 signalling-dependent stimulation of endothelial cell proliferation and tumour vascularization promoted by ASCs indicates angiogenesis as a key underlying mechanism. This finding is consistent with the pro-angiogenic functions reported for CXCL1 and CXCL8 in several different types of cancer^60^. However, CXCL1 signalling activating CXCR1 and CXCR2 in various cell types also causes other effects that may be cancer type and stage dependent^61^. Indeed, a network of paracrine signals between carcinoma, myeloid and endothelial cells that drives both cancer progression and chemoresistance depends on CXCL1 expression by cancer cells^62^. In future studies, it may be important to dissect the direct effects of CXCL1/CXCL8 signalling from the indirect effects of cells recruited by these chemokines.

Our study reveals αSMA as a functional link between CXCL1 signalling and increased tumour vascularization on ASC recruitment. The dynamics of CD34 and αSMA expression in tumour stroma infiltrated by ASCs has been previously reported as an indicator of cancer aggressiveness^52^. The CXCL1–CXCR1/CXCR2 signalling-dependent induction of αSMA in ASCs observed in our studies is consistent with the previous report of αSMA expression being downstream of CXCL8 signalling^63^. αSMA expression on the tumour-recruited CXCR6+ stromal cells was also identified as an attribute responsible for their stimulatory effect on cancer progression^13^. It has been shown that αSMA+ myofibroblasts promote tumour growth^6^. Moreover, we previously showed that αSMA+ myofibroblasts, which also express CD13, function as promoters of tumour vascularization^64^. Acquisition of myofibroblast properties by ASCs has been uncovered as a key component of their pro-angiogenic effects^65^, which likely explains their stimulatory effects on tumour vascularization that we are reporting.

In summary, this study identifies a mechanism through which excess WAT contributes to prostate stroma composition and cancer progression. Like bone-marrow-derived MSCs, ASCs have shown promise in regenerative medicine due to their trophic, angiogenic and immunosuppressive functions. However, their engagement in tumour microenvironment demonstrated recently^34^ puts the safety of lipotransfer procedures in cancer patients under question^66^. At this point, it is not clear if mobilization of ASCs observed in obese patients is a step in their trafficking to the tumour. Studies in breast cancer suggest that ASCs are recruited by tumours from the surrounding WAT^67^. It is likely that PP WAT similarly serves as a source of ASCs in prostate cancer. However, CXCR1 is expressed in ASCs in all WAT depots and our studies in mouse models demonstrated that ASCs can also traffic to tumours from distant WAT through the circulation^27^,^29^. In the future, development of approaches to inactivation of ASC trafficking and their vasculogenic function will be important to assess ASCs as a potential cancer drug target.

## Methods

### Patient specimens

The clinical protocol was approved by MD Anderson Institutional Review Board. See Supplementary Fig. 1 for the demographic and clinical data. The stage of cancer was defined according to the criteria of the AJCC/UICC. For each subject, the BMI (kg m^−2^) was calculated; obese was defined as BMI≥30, non-obese as BMI<30, overweight as BMI 25–30 and lean as BMI<25. For peripheral blood analysis, PBMC samples previously collected (before surgical or other therapeutic interventions) by the University of Texas CTSA Biobank Consortium were used. The cancer-free donors (controls) were individuals 23–78 years of age and BMI of 17.9–29.8 with no prior history of cancer or metabolic disease^31^. Tumour and WAT specimens were from MD Anderson Cancer Center patients with clinically localized prostate cancer selected based on BMI. No matching based on the clinical or pathological stage was performed. Samples of tumour, PP WAT and SC WAT tissues were obtained at the time of prostatectomy. Informed consent was obtained from all subjects. Median follow-up was 3 years. A tissue array HPro-Ade96Sur-01s containing duplicated cores of 36 cancers (8 of which with matched normal adjacent tissue) and 3 cases of metastasis in bones, and 1 in abdominal wall with the follow-up data was purchased from Biomax. Malignant cells were blindly typed by a pathologist based on haematoxylin and eosin (H&E) staining of tissue sections.

### Animal experiments

Mouse studies were approved by the University of Texas Health Science Center Animal Care and Use Committee. Six-week-old C57BL/6 male mice (Stock 000664) were purchased from Jackson laboratory and raised on 58 kcal% (fat) diet (D12331) or chow (as indicated) for 4 months before experiments. Six-week-old Foxn1^nu^ (termed Nude) male mice (Stock 490) were purchased from Charles River. For tumour grafting, 1 × 10^5^ cells were injected with a 21-G needle onto lower back. The tumour size was measured with a calliper, and the volume was calculated as length × width^2^ × 0.52. For ASC-homing experiments, after tumours grew to ∼0.5 cm^3^, 5 × 10^5^ ASCs were SC injected into a contra-lateral flank, and tumours were recovered for analysis the next day. Tissues were recovered from Avertin-anaesthetized mice. Reparixin was injected at 5 mg kg^−1^ SC 5 days per week since the day of tumour grafting. PBS injections were used as a placebo control. Animal groups were coded for tumour analysis to be performed blindly.

### Cell lines and primary cell culture

Cell lines bEnd.3, B16F10 and LLC were obtained from the American Type Culture Collection. 4T1.2 cells were received from R. Anderson, E0771—from F.M. Sirotnak, ID8—from F.C. Marini and RM1—from T.C. Thompson. Primary ASCs and BM MSCs were isolated as described^68^ by digesting minced WAT or bone marrow with collagenase and dispase, passing the preparations through a 40-μm mesh and centrifuging at 400*g* for 8 min to collect the stromal/vascular fraction (SVF) pellet. Immortalized mouse ASCs, previously isolated from a progeny of the cross between H-2K(b)-tsA58 mice and mice ubiquitously expressing GFP (Immorto/GFP)^29^, were cultured at 33 °C. For the assessment of ASC contact-dependent proliferation, admixes of 5 × 10^3^ immortalized mouse ASCs (GFP+) and 5 × 10^2^ RM1 or bEnd.3 cells were plated in culture, detached by trypsin every 2 days, fixed in 4% paraformaldehyde and analysed by flow cytometry. To generate conditioned medium, 5 × 10^6^ cells were seeded into 100-mm plates, cultured with 6 ml α-minimum essential medium/0.5% fetal bovine serum (FBS) for 24 h. For adipogenic differentiation, confluent cells in 24-well plates were cultured in adipogenic induction medium: DMEM (Mediatech) supplemented with 10% FBS, 1% penicillin/streptomycin, 10 μg ml^−1^ insulin, 500 μM 3-isobutyl-1-methylxanthine (Sigma-Aldrich), 1 μM dexamethasone (Sigma-Aldrich) and 200 μM indomethacin (Sigma-Aldrich). After cells were maintained in induction medium for 72 h, the medium was changed to adipogenic maintenance medium: DMEM supplemented with 10% FBS, 1% penicillin/streptomycin and 10 μg ml^−1^ insulin. The maintenance medium was changed two times per week during 10 days of incubation. Cells were then fixed in 10% formalin for 1–2 h, followed by 60% isopropanol. Oil red O (Sigma-Aldrich) staining was applied to visualize red lipid droplets. For osteogenic differentiation, confluent cells in six-well plates were cultured in NH OsteoDiff Medium (Miltenyi) for 3 weeks, with the medium changed twice a week. After 3 weeks, cells were washed with PBS, fixed with 100% methanol and then Von Kossa staining was performed. For chondrogenic differentiation, 5 × 10^5^ cells were centrifuged at 1,200 r.p.m. for 5 min in a 15-ml conical tube with regular culture medium without FBS and resuspend in chondrocyte media: DMEM containing 1% penicillin/streptomycin with L-glutamine, 50 μg ml^−1^ ascorbic acid, 100 nM dexamethasone and 10 ng ml^−1^ TGF-B3. Cell pellets were cultured in a 15-ml conical tube for 21 days, and the medium was changed every 2 days. Pellets were fixed in 10% formalin, and toluidine blue staining was performed to assess chondrogenesis.

### Chemokines and chemokine receptor inhibitors

Recombinant CXCL1 was purchased from R&D Systems Inc. Reparixin was purchased from American Custom Chemicals Corporation. CXCR-blocking antibodies reported previously^69^ from Abcam were used at 2 μg ml^−1^. CXCL1 concentration was determined by ELISA according to the manufacturer's protocol (R&D Systems Inc). All assays were performed independently three times. Neutralizing mouse monoclonal CXCL1 and CXCL8 antibodies (R&D Systems) were used at 1:1,000.

### Gene silencing

Gene-silencing experiments were performed with lentiviral pLKO.1-puro vectors from Sigma-Aldrich based on the reported methodology^49^. Lentiviral vectors (Thermo Scientific) were as follows: sh-CXCL1 (TRCN0000067210–TRCN0000067212), sh-CXCR1 (V2LMM_91345–V3LMM_441730) and sh-CXCR2 (V2LMM_64429–V2LMM_67096). PLKO.1 and PGIPZ vectors with untargeted sequences were used as negative controls shRNA control. RM1 cells and ASCs are infected with lentivirus MOI (multiplicity of infection) 10:1 for 3 days before puromycin selection. The knockdown efficiencies were quantified by RT–qPCR, ELISA and western blotting.

### Immunofluorescence

Formalin-fixed paraffin-embedded tissues were sectioned and analysed by immunofluorescence as described^29^,^70^. Anti-CXCL1 antibodies were goat sc-1374 from Santa Cruz Biotechnology (1:250) and rabbit from Bioss (1:200) with similar results. Other antibodies were as follows: rabbit anti-CXCL8 (Bioss, 1:200); rabbit anti-CXCR1 (GeneTex, 1:300); rabbit anti-CXCR2 (GeneTex, 1:50); rat anti-CD34 (Abcam, 1:100); rat anti-CD45 (eBioscience, 1:100); rabbit anti-PDGFRβ ab32570 (Abcam, 1:100); goat anti-CD31 (Santa Cruz Biotechnology, 1:100); mouse anti-αSMA (Sigma, 1:1,000); and rabbit anti-Ki-67 (Thermo Scientific, 1:100). Donkey Alexa 488-conjugated (1:150) IgG was from Invitrogen and Cy3-conjugated (1:300) IgG was from Jackson ImmunoResearch. All antibodies used were chosen based on the technique that was validated for it by the manufacturer. For CXCL1 and CXCL8, two independently generated antibodies were used with similar results. Nuclei were visualized with 4,6-diamidino-2-phenylindole staining. Isolectin B4 (b1205) was from Vector. CXCL1 and CXCL8 expression in the epithelium and CXCR1 expression in the stroma were scored as (+) when the malignant area contained the majority of cells with signal visibly above that observed in the non-malignant prostate area on the same slide. Malignant and non-malignant prostate areas in tumour sections were identified by a trained pathologist based on H&E staining, and protein expression in those areas was blindly scored by three independent investigators. Immunofluorescence images were acquired at × 20 with Carl Zeiss upright Apotome Axio Imager Z1/ZEN2 Core Imaging software. H&E-staining images and cell images were acquired with an Olympus IX70 inverted fluorescence microscope/cellSens software. For quantifications, at least 10 random × 10 magnification fields were blindly scored and/or measured using microscope grid. Amira 5.4 software (VSG) was used for data capture and analysis.

### *Ex vivo* cell assays

*In vitro* cell migration of ASCs was assessed by a 96-transwell assay (Corning). ASCs (2 × 10^4^ per well) were seeded into the upper chamber. α-MEM medium/0.5% FBS was used as a negative control and α-MEM medium/10% FBS as a positive normalization control. Cells were incubated for 20 h at 37 °C in 5% CO_2_. Non-migrated cells were scraped from the upper surface of the membrane with a cotton swab, and migrated cells remaining on the bottom surface were counted after staining with crystal violet. For proliferation assay, sh-control RM1 and sh-CXCL1 RM1 cells were seeded at 1 × 10^3^ cells per well and cultured in the absence or presence of indicated agents. After 1, 2, 3 and 6 days, RM1 cell proliferations were assessed by Celltiter Blue assay (Promega) using multi-functional microplate reader Infinite 1,000 (Tecan). Assays were performed independently at least three times. Three-lineage differentiation of obese patient PBMC-derived stromal cells with dexamethasone, indomethacin and insulin (adipogenesis); ascorbic acid, β-glycerophosphate and dexamethasone (osteogenesis); or insulin, ascorbic acid and TGF-β for (chondrogenesis), and subsequent staining with Oil red O (adipogenesis), Von Kossa (osteogenesis) and toluidine blue (chondrogenesis) was performed using the established protocols^30^,^53^.

### *In vivo* cell-homing assay

For ASC homing, mice were SC inoculated with 5 × 10^5^ sh-control RM1 and sh-CXCL1 RM1 cells into upper right flank. When tumours grew to ∼0.5 cm^3^, 5 × 10^5^ GFP-expressing sh-control and sh-CXCR1/2 ASCs were SC injected into lower left flank. Tumours were collected after 6 h. Tissue was split for flow cytometry and frozen-section analysis to quantify GFP-positive cells.

### Quantitative RT–PCR

Total RNA was extracted using the Trizol kit (Life Technologies). Complementary DNAs were generated using Superscript III (Life Technologies). PCR reactions were performed on ABI7900 (Applied Biosystems) and TaqMan/Sybregreen Universal PCR Master Mix (Life Technologies). Reactions were run in triplicate using ABI 7,900 workstation and SDS 2.4 software (Applied BioSystems). Expression of CXCL1, CXCR1 and CXCR2 was normalized to 18S RNA. The following TaqMan primers from Life Technologies were used: human CXCR1: HS00174146_m1; human CXCR2: Hs00174304_m1; mouse CXCR1: mm00731329-S1; mouse CXCR2: Mm99999117-S1; and 18S RNA: 4319413e.

### Flow cytometry

Cells were analysed using LSR-II, and populations were isolated by fluorescence-activated cell sorting (FACS) using FACS Aria and FACS Diva software (BD Biosciences). Cells were pre-gated to exclude debris, cell clumps, as well as dead cells based on 4,6-diamidino-2-phenylindole staining. For human cell analysis, fluorescein isothiocyanate-conjugated CD31 antibody (clone WM59), phycoerythrin-conjugated CD34 antibody (clone 8G12) and allophycocyanin-Cy7-conjugated CD45 antibody (clone HI30) along with appropriate isotype IgG controls (BD Bioscience) were used. ASC gating was performed as described^31^ using patient WAT-derived cells as control. Mouse tumour tissue cell suspensions were analysed as described^27^,^68^ based on fluorescence or using the following IgG clones: APC-anti-CD34 (RAM34) or PE-anti-CD34 (MEC 14.7), PE-Cy7-anti-CD31 (390) or PE-anti-CD31 (MEC 13.3), APC-Cy7-CD45 (30-F11), CY5.5-anti-Sca-1 (D7), FITC-anti-CXCR1 (orb15459, Biorbyt), PerCP-CXCR2 (FAB2164C, R&D) and the corresponding isotype controls (BD Biosciences).

### Statistical analysis

Microsoft Excel was used to graph the data as mean±s.e.m. and to calculate *P* values. For comparison of circulating human cell frequencies, which were not normally distributed as determined by the Kolmogorov–Smirnov test, nonparametric Mann–Whitney *U*-test was used. For other analyses, homoscedastic Student's *t*-test was used. *P*<0.05 was considered significant. Sample size calculations were performed for 80% power. No exclusions were done. No randomization was used. The variance was similar for the animal data. All experiments were repeated at least twice (the majority three times) with similar results.

### Data availability

The data that support the findings of this study are available from the corresponding author on request.

## Additional information

**How to cite this article:** Zhang, T. *et al*. CXCL1 mediates obesity-associated adipose stromal cell trafficking and function in the tumour microenvironment. *Nat. Commun.* 7:11674 doi: 10.1038/ncomms11674 (2016).

## Supplementary Material

Supplementary Information Supplementary Figures 1-7
