# Supplementary material for: CXCL1 mediates obesity-associated adipose stromal cell trafficking and function in the tumour microenvironment
Source: Nat Commun. 2016 May 31;7:11674. doi: 10.1038/ncomms11674 (PMC4895055; doi:10.1038/ncomms11674)
Supplement: Supplementary Information — Supplementary Figures 1-7 [file ncomms11674-s1.pdf]

a

Patient ID	BMI	Age	Gleason score	Patient ID	BMI	Age	Gleason score
Non-obese				*Obese*			
PBMC 1	22.1	81	6 (3+3)	PBMC 25	30.5	73	N/A
PBMC 2	21.9	65	6 (3+3)	PBMC 26	50.9	60	7 (4+3)
PBMC 3	22	84	8 (4+4)	PBMC 27	34.3	68	N/A
PBMC 4	24.6	68	7 (3+4)	PBMC 28	31.8	52	N/A
PBMC 5	24.5	55	6 (3+3)	PBMC 29	30.6	58	N/A
PBMC 6	24.7	73	7 (3+4)	PBMC 30	31	N/A	N/A
PBMC 7	23.5	67	7 (3+4)	PBMC 31	30.6	47	7 (3+4)
PBMC 8	23.7	N/A	N/A	PBMC 32	30.8	66	9 (4+5)
PBMC 9	23.7	65	7 (4+3)	PBMC 33	31.2	70	8 (4+4)
PBMC 10	23.2	52	6 (3+3)	PBMC 34	41.2	62	7 (4+3)
PBMC 11	24.4	65	6 (3+3)	PBMC 35	30.2	70	6 (3+3)
PBMC 12	23.7	56	N/A	PBMC 36	31	63	9 (5+4)
PBMC 13	22.5	56	9 (4+5)	PBMC 37	40.7	57	N/A
PBMC 14	23.7	80	9 (5+4)	PBMC 38	34.8	63	7 (3+4)
PBMC 15	23.3	76	10 (5+5)	PBMC 39	31.5	63	8 (4+4)
PBMC 16	24.6	48	9 (4+5)	PBMC 40	32.6	64	10 (5+5)
PBMC 17	23.6	66	7 (3+4)	PBMC 42	38.9	53	8 (4+4)
PBMC 18	23.2	73	7 (3+4)	PBMC 42	40.6	63	N/A
PBMC 19	24.5	86	8 (3+5)	PBMC 43	32.6	65	N/A
PBMC 20	20.4	64	8 (4+4)	PBMC 44	32.6	74	7 (4+3)
PBMC 21	23.6	62	7	PBMC 45	31	63	N/A
PBMC 22	20.1	60	6 (3+3)				
PBMC 23	22.7	68	7 (4+3)				
PBMC 24	23.1	52	6 (3+3)				

b

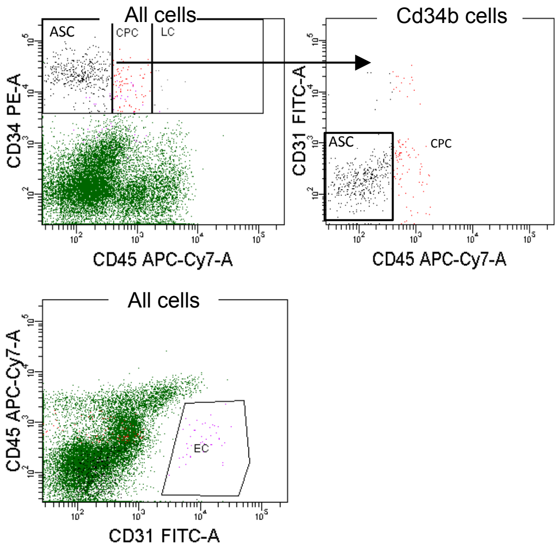

c

Patient ID	BMI	CXCL1	CXCR1	CXCL8	Clinical Stage	Pathologic Stage	Lymph node Stage	Gleason Score	Tumor volume (cc)	Age	Race	Progression
Non-obese												
P 1	27.8	(-)	(-)	(-)	T1c	pT2	pN0	7 (3+4)	0.48	60	W	
P 7	28.2	(-)	(-)	(-)	T1c	pT2	pN0	7 (4+3), 5	0.32	58	W	
P 13	25.6	(-)	(+)	(+)	T2c	pT3a	pN0	7 (4+3), 5	0.62	67	A	
P 18	29.9	(-)	(+)	(+)	T1c	pT2	pN0	7 (4+3)	0.14	76	W	
P 35	29.2	(-)	(-)	(+)	T2a	pT2	pN0	6 (3+3)	0.26	58	B	
P 36	27.5	(-)	(-)	(+)	T1c	pT3a	pN0	7 (3+4)	6.03	61	B	
P 37	25	(-)	(-)	(+)	T2c	pT3b	pN0	7 (3+4)	0.44	67	W	
P 39	28.9	(-)	(-)	(-)	T2b	pT3b	pN0	7 (4+3)	1.77	71	W	
P 41	27.5	(-)	(-)	(+)	T2a	pT2	pN0	7 (3+4)	1.97	42	W	
P 42	28.5	(+)	(-)	(-)	T1c	pT2	pN0	7 (3+4)	1.09	67	W	
P 43	23.6	(-)	(+)	(+)	T2c	pT3a	pN0	8 (4+4)	2.03	59	A	
P 44	27.9	(-)	(+)	(+)	T1c	pT2	pN0	9 (4+5)	0.44	63	W	
P 45	28.7	(-)	(-)	(-)	T1c	pT3a	pN0	7 (3+4)	0.92	49	W	
P 46	27.5	(-)	(-)	(+)	T2b	pT3a	pN0	9 (4+5)	1.00	58	W	
P 47	28.8	(-)	(-)	(-)	T1c	pT2	pN0	8 (4+4)	0.34	57	B	
P 48	28.4	(-)	N/A	(-)	T1c	pT2	pN0	7 (4+3), 5	1.34	64	W	
Obese												
P 4	31.6	(+)	(+)	(-)	T2a	pT2	pN0	7 (3+4)	6.24	66	W	
P 5	30.3	(+)	(-)	(-)	T1c	pT2	pN0	7 (3+4)	0.44	56	W	Biochemical recurrence
P 6	31.5	(+)	(-)	(-)	T1c	pT2	pN0	7 (3+4)	0.24	74	W	
P 8	31	(+)	(+)	(-)	T2a	pT2	pNx	7 (3+4)	0.42	66	A	
P 10	43.9	(+)	(-)	(+)	T3b	pT3b	pN1	9 (4+5)	15.23	65	W	
P 11	32.5	(+)	(+)	(+)	T3a	pT3a	pN0	9 (4+5)	3.97	60	H	Biochemical recurrence
P 12	33.5	(+)	(+)	(+)	T3a	pT2	pN0	9 (4+5)	2.80	67	W	
P 14	43.4	(+)	N/A	(+)	T1c	pT2	pN0	7 (3+4)	0.21	63	W	
P 16	34.9	(+)	(+)	(+)	T1c	pT2	pNx	7 (3+4)	0.20	61	W	
P 17	31	(+)	(+)	(+)	T1c	pT2	pNx	7 (3+4)	0.56	51	W	
P 19	36.7	(-)	(+)	(+)	T1c	pT2	pNx	7 (3+4)	2.41	66	W	
P 20	37.4	(+)	(+)	(-)	T1c	pT2	pNx	7 (3+4)	0.59	42	W	
P 21	38.6	(+)	(+)	(+)	T3b	pT3b	pN1	9 (4+5)	6.00	53	H	Death
P 23	35.8	(+)	(+)	(+)	T2b	pT2	pNx	7 (3+4)	0.96	64	W	
P 25	31.9	(+)	(+)	(-)	T1c	pT2	pN0	7 (3+4)	0.19	54	W	
P 28	32.6	(+)	(+)	(-)	T1c	pT2	pN0	7 (3+4)	0.03	53	W	
P 30	31.5	(+)	(+)	(-)	T2a	pT2	pN1	7 (3+4)	2.00	53	B	
P 31	30	(-)	(+)	(-)	T2a	pT2	pN0	7 (3+4)	1.71	62	B	
P 32	38.3	(+)	(+)	(+)	T2a	pT2	pN0	7 (3+4)	4.48	61	W	
P 33	30.6	(+)	(+)	(+)	T1c	pT2	pN0	7 (3+4)	0.75	57	B	
P 34	32.3	(+)	(+)	(-)	T2c	pT2	pN0	7 (3+4)	0.11	42	B	
P 3	39.8	N/A	N/A	N/A	T2b	pT3a	pN0	8 (4+4)	1.36	53	W	Local recurrence
P24	34.1	N/A	N/A	N/A	T1c	pT3b	pN1	8 (4+4)	3.97	47	W	Biochemical recurrence

Supplementary Figure 1 | Characteristics of PC patients. (**a**) Patients who's PBMC were analyzed in Fig. 1a. (**b**) Flow cytometric gating of cells from a periprostatic (PP) WAT sample identifying CD34bCD45-CD31- ASC, CD34bCD45b leukocytes (LC), CD34dCD31bCD45- EC, and CD34bCD31dCD45d CPC. b, bright, d, dim. Data are representative of three independent experiments. (**c**) Patients who's tissues were analyzed in Fig. 2a-c and Supplementary Fig. 2a-d,f. CXCL1 and CXCL8 expression in epithelium and CXCR1 expression in the stroma is scored as high (+) and low (-). N/A: data not available. pN1: pelvic lymph node metastasis. W: white, B: black, H: hispanic; A: asian.

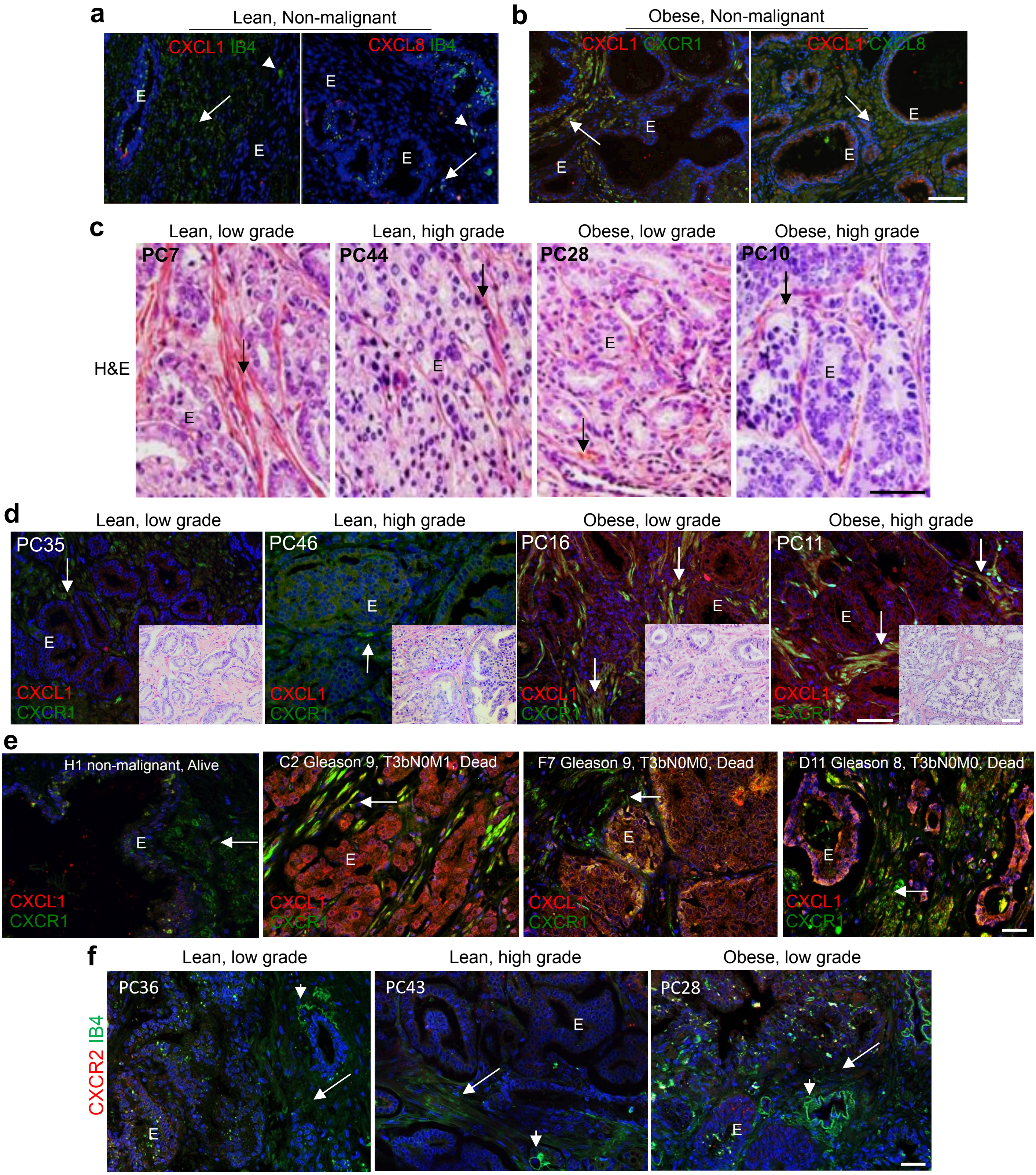

Supplementary Figure 2 | The CXCL-CXCR obesity axis. (**a-b**) IF analysis of non-malignant prostate areas of tumors from lean (**a**) and obese (**b**) patients with CXCR1 or CXCL8 antibodies (red) counter-stained with CXCR1 or CXCL8 antibodies or IB4 (green). (**c**) Hematoxylin/eosin (H/E) stainings of sections analyzed in Fig. 2a. (**d**) IF analysis of tumors from additional indicated patients with CXCL1 (red) and CXCR1 (green) antibodies. Insets: H/E staining of the area. (**e**) IF analysis of indicated cores from Biomax array HPro-Ade96Sur-01s with CXCL1 antibodies (red) and CXCR1 antibodies (green). (**f**) IF analysis of indicated human prostate tumor sections with CXCR2 antibodies (red) and endothelium-labeling IB4 (green). Arrowheads: endothelium. Arrows: stroma, E: epithelium. Nuclei are blue. Scale bar (**a-f**): 100 μm. Data are representative of three independent experiments in **a-d** and **f**.

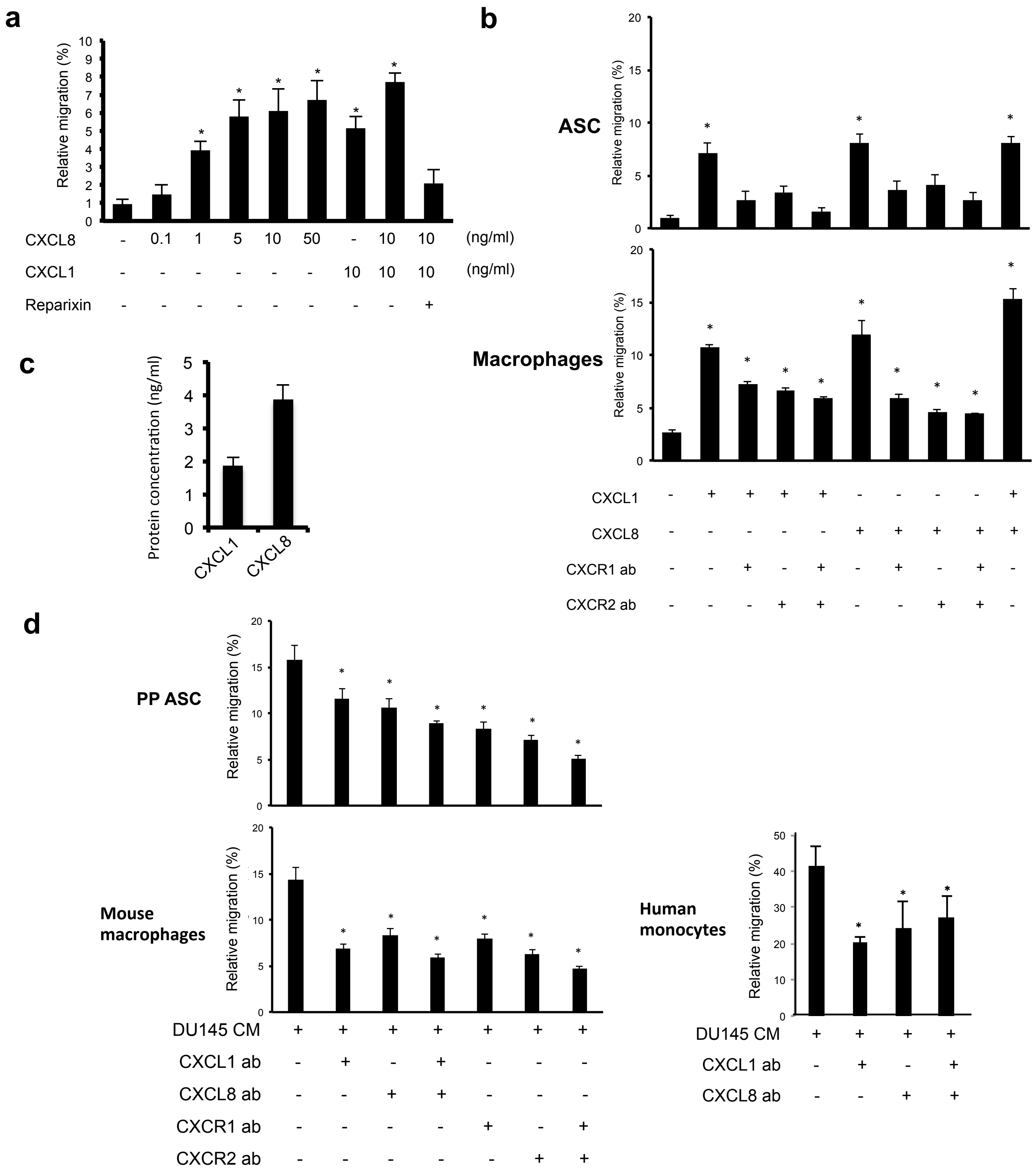

Supplementary Figure 3 | Chemotaxis toward CXCL1 and CXCL8. (**a**) Human PP ASC were subjected to trans-well chamber migration through 8 μm pores toward serum-free medium (-) supplemented (+) with increased indicated concentrations of CXCL1 and / or CXCL8 in the presence of reparixin where indicated. (**b**) Human ASC and immortalized mouse macrophages were subjected to migration toward serum-free medium (-) supplemented (+) with 10-20 ng/ml of CXCL1 or CXCL8 and neutralizing antibodies (ab) blocking CXCR1 or CXCR2. (**c**) CXCL1 and CXCL8 concentration in DU145 conditioned medium (CM) measured by ELISA. (**d**) Human ASC, mouse macrophages and primary human PBMC-derived monocytes were subjected to migration toward DU145 CM. Where indicated CM was supplemented with neutralizing ab against CXCL1, CXCL8, CXCR1 or CXCR2. Plotted are relative numbers normalized to migration of cells towards 20% FBS set as 100%. Relative cell migration in the absence of CM was < 5%. *$p<0.05$ vs no-chemokine or no-antibody controls (Student's t-Test). In all panels, graphs show mean ± s.e.m. for technical triplicates.

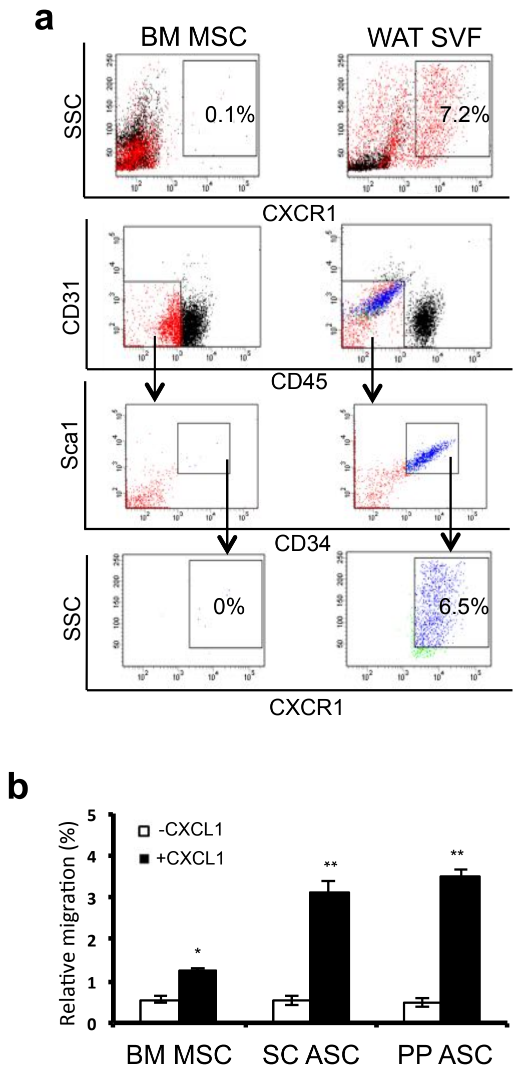

Supplementary Figure 4 | Bone marrow MSC are not subject to the CXCL1-CXCR1 signaling. (**a**) A representative flow cytometric analysis of MSC from mouse bone marrow (BM) and WAT stromal/vascular fraction (SVF) showing the expression of CXCR1 on total and CD45-CD31-CD34+Sca1+ stromal cells. Arrows indicate subsequent gating steps. % of total viable cells is indicated. (**b**) Bone marrow-derived MSC from a cancer-free donor and ASC isolated from PP WAT of a non-obese prostate cancer patient were subjected to trans-well chamber migration toward medium with or without 5 ng/ml CXCL1. Plotted is relative migrated cells numbers normalized to migration of SC ASC toward 10% FBS set at 100%. Data represent mean ± s.e.m. for technical triplicates; **P*<0.05; ** *P*<0.001 (Student's t-Test) versus control (no CXCL1).

a

Analyte	Obese / Lean
G-CSF	2.00
Eotaxin	1.71
GM-CSF	-
IFN-r	-
IL-1a	0.96
IL-1b	0.52
IL-2	-
IL-4	-
IL-3	-
IL-5	0.85
IL-6	0.92
IL-7	0.66
IL-9	2.30
IL-10	1.02
IL-12p40	1.09
IL-12p70	0.22
LIF	-
IL-13	0.60
LIX	0.93
IL-15	1.27
IL-17	-
IP-10	0.84
**CXCL1 (KC)**	2.30
MCP-1	0.19
MIP-1a	0.48

Analyte	Obese / Lean
MIP-1b	-
MCSF	0.06
MIP-2	0.48
MIG	0.46
RANTES	0.86
VEGF	-
TNFa	-
EPO	0.91
Exodus-2	0.76
MCP-5	0.86
MIP-3b	0.76
MIP-3a	0.72
TARC	0.60
IL-16	1.34
Fractalkine	0.88
IL-21	-
IL-22	48.97
IL-25	1.20
IL-28b	1.12
MDC	0.62
IL-23	0.12
IL-27	0.13
TIMP-1	1.24
IL-20	-
IL-33	-

b

```
                                                   CXCR1/2-sh
                                              CCCTGCCCATCTTAATTCT
*CXCR1*  641 CGACCTACTCTTTTCATTGACCCTGCCCTTCTTGGCTGTCTCCAAATTGAAGGGCT ...
                                                   CTTCTTGGCTGTCTCCAAA
                                                        CXCR1-sh

                                                          CXCR1/2-sh
                                                   CCCTGCCCATCTTAATTCT
*CXCR2*  642 CATGTGGTTACTATCAGTAATTCTGGCCCTGCCCATCTTAATTCTACGAAATCCT ...
```

c

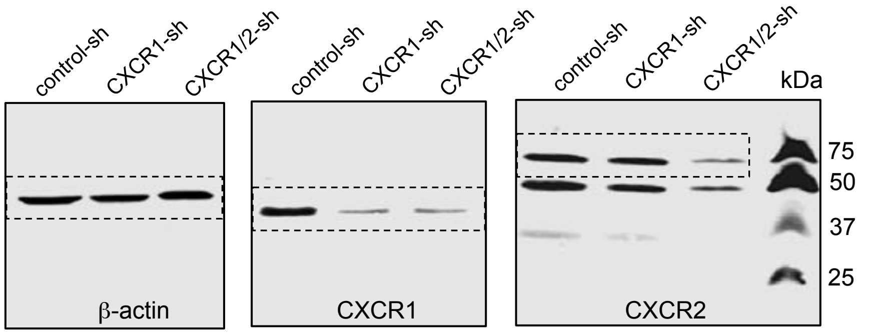

Supplementary Figure 5 | Mouse chemokine and chemokine receptor analysis. (**a**) Activation of mouse CXCL1 in obesity / cancer. Plasma samples were collected from mice (n=5/group) 11 weeks after subcutaneous ID8 cell grafting in to lean or obese mice. For each cytokine analyzed, presented are ratios of the mean plasma concentrations found for the indicated compared mouse groups. Highlighted are the Luminex panel analytes increased more than 2-fold in plasma of obese animals with tumors compared to lean animals with tumors. (-) analytes were undetectable in circulation. (**b**) Alignment of mouse *CXCR1* and *CXCR2* sequences with shRNAs. Gene sequence alignment (starting at indicated nucleotide) shows the areas targeted by CXCR1-sh and CXCR1/2-sh. (**c**) Uncropped scans of the Western blotting (performed twice) shown in Fig. 5a.

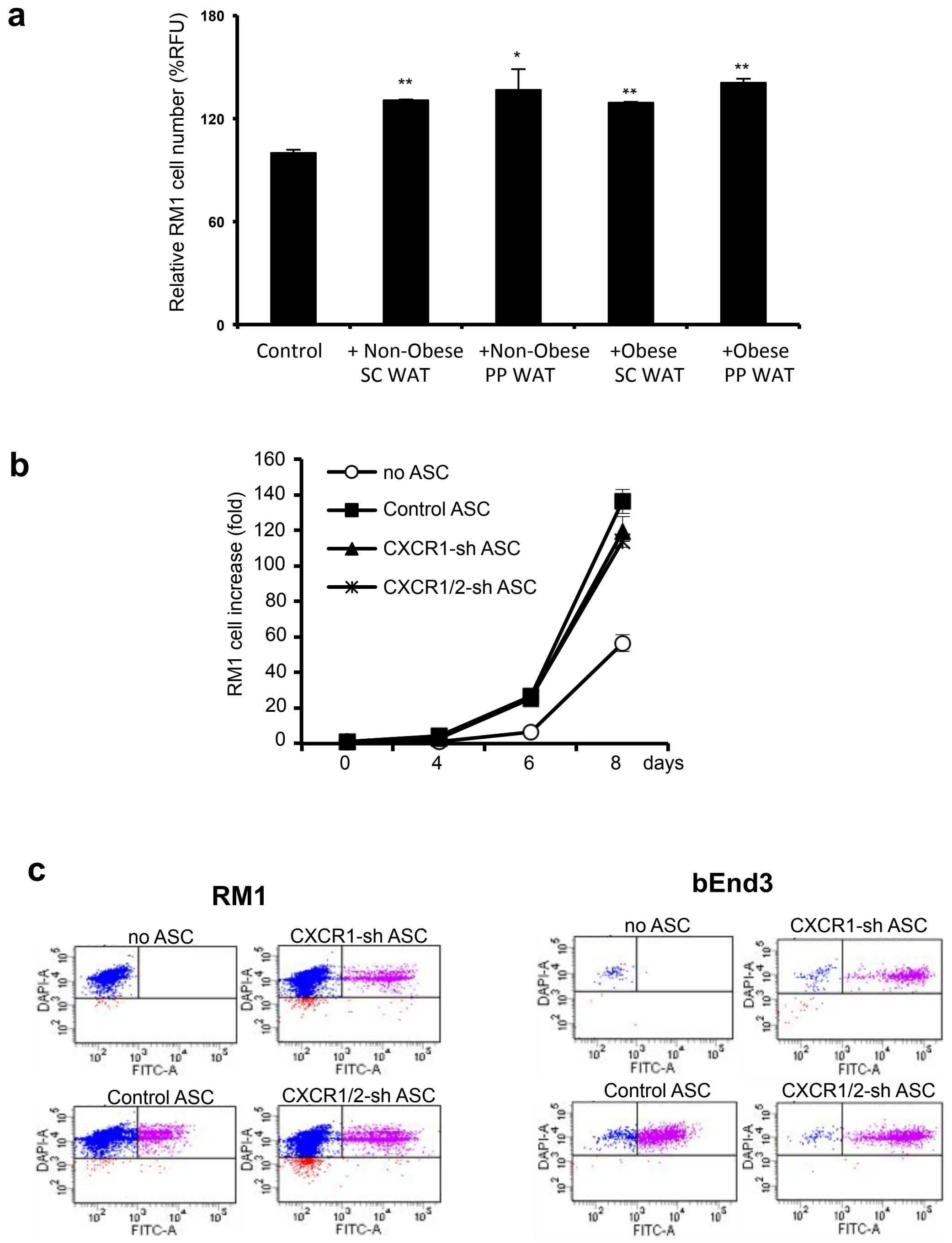

Supplementary Figure 6 | The effect of ASC on cancer cell growth. (**a**) Proliferation (assessed by MTT assay) of RM1 cells in control medium (no ASC) or in medium conditioned by ASC isolated from SC and PP WAT of obese and non-obese prostate cancer patients. Data represent mean ± s.e.m. for technical triplicates; *P<0.05; ** P<0.001 (Student's t-Test) versus control (no WAT). (**b**) Proliferation of RM1 cells in direct co-culture with GFP-labeled immortalized mouse ASC transduced with control-sh, CXCR1-sh or CXCR1/2-sh. Plotted are numbers of bEnd.3 (FITC-) cells collected after the indicated numbers of days. Data represent mean ± s.e.m. for technical triplicates. (**c**) Proliferation of RM1 and bEnd3 (FITC-) cells in direct co-culture with GFP-labeled immortalized mouse ASC (FITC+) transduced with control-sh, CXCR1-sh or CXCR1/2-sh. Flow cytometry data post-fixation at day 8 of co-culture are shown. Data are representative of three independent experiments.

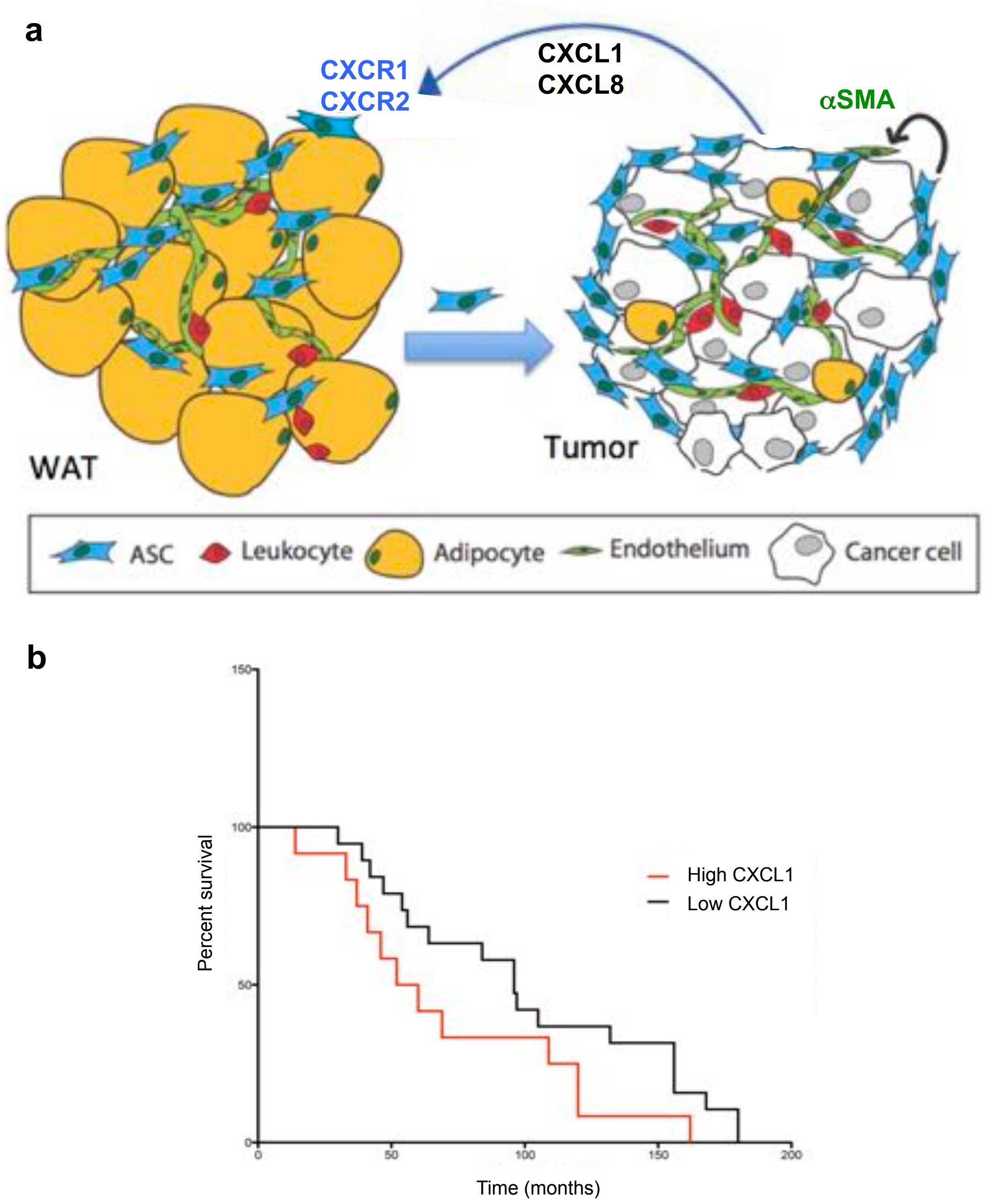

Supplementary Figure 7 | CXCL1/CXCL8 signaling and cancer progression. (a) A working model for mechanism of ASC trafficking in cancer. *In vivo*, ASC express CXCL1 / CXCL8 receptor CXCR1 and possibly CXCR2, expression of which in ASC is increased in cell culture. Obesity-induced secretion of CXCL1 and malignancy-induced secretion of CXCL8 by epithelial cancer cells creates chemokine gradients that enable ASC trafficking to tumors via CXCR1/2. Upon recruitment of ASC to tumors, αSMA expressed downstream of CXCR1 signaling mediates the pro-angiogenic effects of ASC that promote tumor growth. (**b**) A Kaplan–Meier plot showing increased mortality in human prostate cancer patients with high expression of CXCL1 mRNA (Z score >2) in the prostate. Based on data from http://www.cbioportal.org/study.do?cancer_study_id=prad_mich.
